# *Drosophila* and mouse intestinal stem cells are spatiotemporally specified by Notch suppression and Wnt activation

**DOI:** 10.1126/sciadv.ady7272

**Published:** 2025-12-03

**Authors:** You Wu, Lingxiao Yu, Yuxin Yu, Song Wu, Qili Yuan, Weicheng Duan, Sicheng Cai, Bo Xiong, Rong Lin, Zheng Guo

**Affiliations:** ^1^ Department of Medical Genetics, School of Basic Medicine, Institute for Brain Research, Tongji Medical College, Huazhong University of Science and Technology, Wuhan 430022, China.; ^2^ Department of Geriatrics, Union Hospital, Tongji Medical College, Huazhong University of Science and Technology, Wuhan 430022, China.; ^3^ Department of Forensic Medicine, Tongji Medical College, Huazhong University of Science and Technology, Wuhan 430030, China.; ^4^ Department of Gastroenterology, Union Hospital, Tongji Medical College, Huazhong University of Science and Technology, Wuhan 430022, China.; ^5^ Cell Architecture Research Center, Huazhong University of Science and Technology, Wuhan 430030, China.

## Abstract

The specification of intestinal stem cells (ISCs) during development is critical for maintaining intestinal homeostasis. However, the mechanisms underlying this process remain elusive. Here, by counting and tracing ISC in *Drosophila* pupal midgut, we show that ISCs are specified within a narrow 12-hour developmental window, with ~150 ISCs emerging from a pool of ~6000 intestinal epithelial cells. Single-cell sequencing revealed the involvement of Notch and Wnt signaling, with genetic experiments demonstrating that ISC specification requires both Notch suppression and Wnt activation. Furthermore, we showed that Wnt signaling is activated in discrete spatial domains, and Notch-mediated lateral inhibition specifies ISCs in these Wnt-active zones, achieving a ratio of ~1/40. Notably, Notch suppression also promoted the specification of Lgr5^+^ progenitors in the mouse embryonic intestine. Together, our data show that Wnt activation defines niches permissive for ISC fate, whereas Notch suppression licenses fate commitment, a spatiotemporal coordination conserved from insects to mammals.

## Teaser

Intestinal stem cells are specified by spatiotemporal teamwork: Notch suppression carves fate while Wnt zones set the stage.

## INTRODUCTION

The intestinal epithelium is a rapidly renewing tissue that relies on intestinal stem cells (ISCs) to maintain homeostasis, repair damage, and adapt to environmental challenges (*1*–*3*). Uncontrolled ISC proliferation drives colorectal cancer (*4*, *5*), while ISC depletion contributes to ulcerative colitis (*6*, *7*), radiation enteropathy (*2*), and age-related epithelial degeneration (*8*). Despite this clinical significance, the mechanisms that govern how ISCs are initially specified from intestinal epithelium and spatially organized during development remain enigmatic. A deeper understanding of how ISCs are specified, positioned, and maintained during development could aid the discovery of precision strategies to manipulate ISCs in disease.

In *Drosophila*, it is known that ISCs are specified from adult midgut progenitor cells (AMPs) during the early pupal stage and that the unspecified AMPs differentiate into enterocytes (ECs) (*9*–*11*), but the detailed process is largely obscure. During embryogenesis, endoblasts in the embryonic endoderm give rise to AMPs (*12*). During larval stages 1 and 2 (L1 and L2), AMPs proliferate and spread throughout the midgut. In the third instar (L3), spreading stops and the founding AMP undergoes asymmetric division to generate a peripheral cell (PC) (*10*), which acts as a niche in which the AMP and its subsequent daughters can remain undifferentiated and proliferate to form an AMP island (*13*). When the larval midgut histolysis occurs at metamorphosis, the PCs break down, allowing all the AMPs to fuse basally to form the de novo pupal midgut epithelium, leaving the rest of the larval epithelium that wraps around the larval food to become meconium in the pupal midgut (*11*, *14*). It has been hypothesized that one AMP per island is preselected to remain undifferentiated and become a future ISC (*10*). However, this hypothesis has not been tested experimentally.

Mammalian adult Lgr5^+^ ISCs are derived from the fetal intestinal epithelium (*15*, *16*). In fetal mice, the primitive gut tube is formed by embryonic day 9 (E9.0) (*1*, *17*). From E9.0 to E13.5, it remains as a smooth lining of the primitive gut tube comprised of morphologically identical epithelial cells (*18*). At stage E13.5, embryonic Lgr5^+^ ISCs emerge at intervals in this undeformed intestinal tube (*17*). The location of their emergence correlates with crypt morphogenesis and is thought to lead to the formation of future crypts at their location by generating an apical constriction (*19*–*21*). Although it has been shown that embryonic ISC specification at stage E13.5 is closely linked to the activation of Wnt signaling (*17*), how ISCs can be specified at intervals requires further investigation.

Here, we reveal a conserved Wnt/Notch signaling axis governing ISC specification. We demonstrate that, in contrast with the AMP preselection model, *Drosophila* ISCs are cooperatively specified by Notch suppression and Wnt activation. Wnt ligands from midgut circular muscles and larval gut remnants (meconium epithelium) establish discrete spatial Wnt-active niches competent for ISC specification. Notch signaling then specifies ISC via lateral inhibition in those Wnt-active zones, ensuring precise ISC patterning. Notch inhibition promoted the specification of Lgr5^+^ progenitors in the mouse embryonic intestine.

## RESULTS

### ISCs are specified at 12.5 hours APF of *Drosophila* pupal midgut

**Fig. 1.**
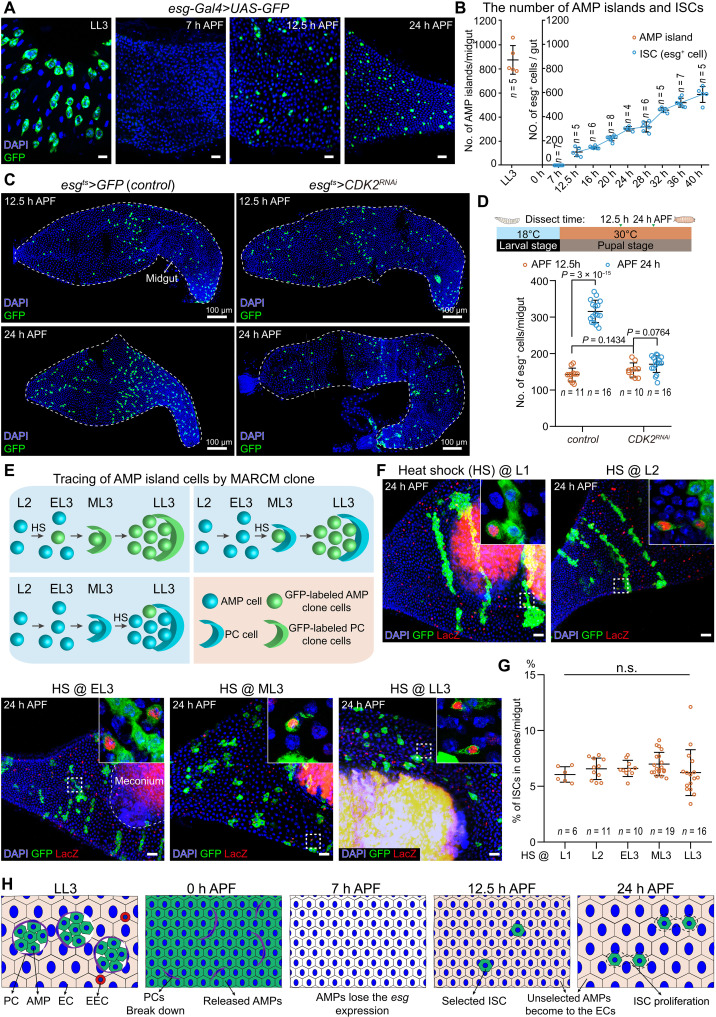
ISCs are specified from *Drosophila* pupal midgut epithelium at 12.5 hours APF. (**A**) Representative confocal images of *esg*>GFP (green)–labeled ISCs in the larval (LL3) and pupal midgut. In all images, cell nuclei are stained with 4′,6-diamidino-2-phenylindole (DAPI; blue). (**B**) Quantification of AMP islands (LL3) and ISCs (*esg*>GFP) at indicated pupal stages. *n*, number of midguts. Trend lines connect mean values. (**C** and **D**) Representative images (C) and statistics of ISC number (D) *control* (*esg^ts^*>*GFP*) and *esg^ts^*>*Cdk2^RNAi^* pupal midguts at 12.5 or 24 hours APF. Schematic in (D): experimental design for the temporal *Cdk2* knockdown in ISCs. White dashed lines outline the boundary of pupal midgut. (**E**) Schematic of larval stage-specific heat shock (HS)–induced MARCM clones contributing to the AMP lineages. Three HS time points are shown (HS before EL3, ML3, and LL3, respectively). (**F**) Representative MARCM clones (HS before L1, L2, EL3, ML3, and LL3) with *esg*-LacZ (red) labeling in pupal midgut at 24 hours APF. *esg*-LacZ–labeled ISCs could be found in all these stage-specific induced MARCM clones. Inset represents close-up views of *esg*-LacZ^+^ clone cells in selected box regions. Autofluorescent meconium is indicated in the midgut. (**G**) Statistics of the ISC percentage (%) in scored MARCM clone cells per midgut. *n*, number of midguts. Not significant (n.s.), *P* > 0.05 by one-way analysis of variance (ANOVA). Clone cells counted in these midguts: L1 1364, L2 1868, EL3 1829, ML3 3623, and LL3 2271. (**H**) Cartoon of the ISC specification process from LL3 AMP islands to 24 hours APF. AMP, adult midgut progenitor; PC, peripheral cell; EC, enterocyte; EEC, enteroendocrine cell. Data are means ± SD. *n*, number of midguts. Statistical analysis by two-tailed unpaired *t* test (D) and one-way ANOVA with Bonferroni’s multiple-comparisons test (G), *P* values are shown in panels. Scale bars, 20 μm unless otherwise specified. h, hours.

To experimentally demonstrate the numerical correlation between AMP islands and ISC specification, we quantified the population dynamics of AMP islands in the *Drosophila* larval midgut and ISCs during early pupal development. Using the AMP specific driver *esg-Gal4* (*22*) to express *UAS-GFP* (*esg*>GFP), we tracked the number of AMP islands at late third instar larval stage (LL3). Because works from our lab and other labs have demonstrated that *esg*>*GFP*^+^ cells are ISCs before 43 hours after puparium formation (APF) (*23*–*26*), we tracked the number of *esg*>*GFP*^+^ cells up to 40 hours APF. At LL3, we identified ~900 AMP islands per midgut containing ~6000 AMP cells (Fig. 1, A and B, and fig. S1A). During the pupal stage, *esg*>GFP was undetectable at 7 hours APF but began to appear in ~150 cells distributed regionally in the midgut at 12.5 hours APF. This population expanded to ~300 cells at 24 hours APF and further increased to ~600 cells at 40 hours APF. To determine whether the increase in ISCs after 12.5 hours APF was due to proliferation or sustained specification, we used a temperature-inducible *esg-Gal4 tub-Gal80^ts^* (*esg^ts^*) system (*22*) to drive RNA interference (RNAi) against *Cdk2*, thereby blocking cell cycle progression (*26*, *27*). Shifting animals to the permissive temperature (30°C) at 0 hours APF revealed that ISC numbers remained static at ~150 cells at 12.5 hours APF and showed no substantial increase by 24 hours APF (Fig. 1, C and D). This suggests that the increase in ISC numbers after 12.5 hours APF was attributable to ISC proliferation. Consistent with this finding, phospho-histone H3 staining–positive (PH3^+^) mitotic cells were detected in the normal 20 hours APF midgut (fig. S1B).

To further rule out the possibility of sustained specification of ISCs after 12.5 hours APF, we ablated all the ISCs specified by 12.5 hours APF by temporally expressing the pro-apoptotic gene *hid* (*28*, *29*) under *esg^ts^* driving. By switching to 18°C from 12.5 hours APF to terminate *hid* expression, our results showed that there was no newly specified ISC in the midgut by 24 hours APF (fig. S1C), confirming that the ISC specification occurs before 12.5 hours APF.

Our quantification revealed a mismatch: The number of AMP islands (~900) in LL3 larval epithelia vastly exceeded the ISC population (~150 cells) specified by 12.5 hours APF (Fig. 1, A and B), excluding one AMP island that contains one ISC hypothesis. To test whether ISC specification correlates temporally with AMP island division, we generated wild-type MARCM (mosaic analysis with repressible cell marker) clones (*30*) at distinct time windows during AMP development (Fig. 1E) and quantified the frequency of ISC specification in the MARCM clones. Notably, the ISC specification rate remained constant (~6% at 24 hours APF) regardless of clone induction timing (Fig. 1, F and G; and fig. S1, D and E), indicating that ISC specification does not depend on the timing of AMP island division and differentiation. Together, our data demonstrate that ~150 ISCs were specified from ~6000 AMPs by 12.5 hours APF in the pupal midgut, and the subsequent ISC symmetric divisions expanded the pool of ISCs (Fig. 1H).

### scRNA-seq reveals the route of ISC specification

**Fig. 2.**
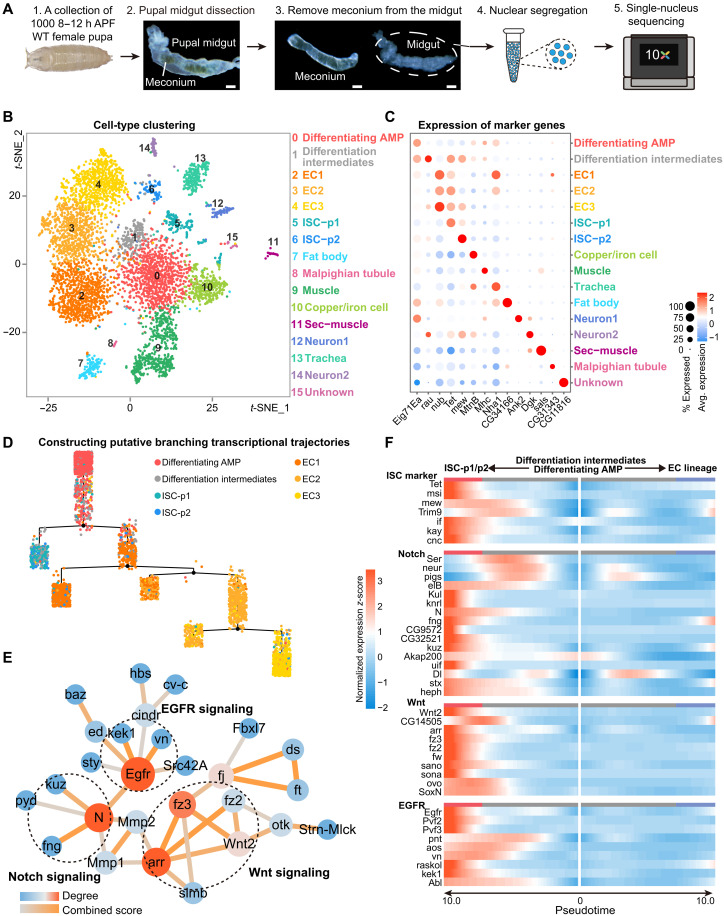
snRNA-seq reveals enrichment of Notch, Wnt, and EGFR signaling genes during ISC-p specification. (**A**) Diagram of the single-nucleus RNA sequencing (snRNA-seq) method for 8 to 12 hours APF pupal midguts. See also Materials and Method. Scale bars, 100 μm. (**B**) *t*-SNE representation of snRNA-seq data colored for cell type based on marker gene expression. Sample size, 5384 nuclei. (**C**) Dot plot depicting marker gene expression across clusters. (**D**) Trajectory tree plot showing the branching of the epithelial cells from differentiating AMPs to ISC-p1/2 and EC1-3. Branches (black lines) indicate differentiation paths, and branch points (nodes) denote transcriptional bifurcations. (**E**) Protein-protein interaction (PPI) network analysis reveals Notch-Wnt-EGFR pathway convergence during ISCp1/p2 specification from differentiating AMP lineages. (**F**) Gene expression heatmap of ISC-p1/2 fate marker and three signaling modules created by clustering the ISC branch in a pseudotemporal order. The middle represents the start pseudotime of AMP differentiating.

To investigate ISC specification mechanisms, we performed single-nucleus RNA sequencing (snRNA-seq) on 1000 meconium-free female pupal midguts collected at 8 to 12 hours APF (Fig. 2A). Our analysis identified 5384 high-quality nuclei that were clustered into 16 distinct groups using the top 30 principal components at a resolution of 0.9 (Seurat v5.0.3), visualized through *t*-SNE (*t*-distributed stochastic neighbor embedding) dimensionality reduction (Fig. 2B). Specific cell identities were assigned on the basis of established markers: fat body (cluster 7), Malpighian tubule (cluster 8), copper/iron cells (cluster 10), and trachea (cluster 13) (Fig. 2C and table S1) (*31*). Clusters 9 and 11 were identified as muscle lineages, while both expressed muscle-specific markers [*Mhc* (*32*) and *sls* (*33*)], cluster 11 uniquely expressed secretion-associated genes [*Ptth* (*34*)], leading to their classification as muscle and secretory muscle, respectively. Neuronal clusters 12 and 14 were defined by neuron-specific markers.

Two ISC progenitor (ISC-p) clusters (5 and 6) were identified through specific expression of ISC markers, with ISC-p1 (cluster 5) highly expressing *Tet* (*31*, *35*, *36*) and ISC-p2 (cluster 6) showing exclusive expression of *mew* (*37*, *38*) and *Trim9* (*31*) (table S1). To further validate *Tet* and *mew* as marker genes for ISC-p1/p2, we performed immunostaining against Tet–enhanced green fluorescent protein (EGFP) (*35*) and Mew (*37*) in the 12.5 hours APF midgut. The results showed clear colocalization of both Tet-EGFP and Mew with esg^+^ cells (fig. S2, A and B), supporting their use as reliable markers for identifying ISC-p1/p2 populations during the pupal stage. Gene Ontology (GO) enrichment analysis further corroborated these ISC-p annotations (fig. S2C and table S2). EC clusters EC1-EC3 (clusters 2 to 4) were identified by expression of *nub* (*Pdm1*) (*39*) and *CG13321* (*31*) (fig. S2D).

Cluster 0 displayed elevated expression of mitochondrial oxidative respiration genes [*ATPsynB* (*40*) and *Cyt-c-p* (*41*)], epidermal morphogenesis markers [*vkg* (*42*) and *Rho1* (*43*)], and ecdysone signaling components [*Eig71Ea* (*44*)] (table S3). This profile matches reported characteristics of differentiating AMPs during ecdysone-triggered metamorphosis (*25*, *45*), which involves increased mitochondrial activity (*46*–*48*). We therefore designated cluster 0 as “differentiating AMPs.” Cluster 1, positioned between differentiating AMPs and differentiated ISC-p/EC clusters, exhibited transitional transcriptional features and was classified as “differentiation intermediates.” Pseudotemporal trajectory analysis (Monocle2) validated our epithelial cluster annotations (0 to 6), with clusters 0 and 1 occupying upstream positions in both ISC-p and EC differentiation trajectories (fig. S2E). Moreover, the expression dynamics of *mew* (an ISC-p marker) and *nub* (an EC marker) along the pseudotemporal trajectory support the accuracy of our cell type annotations (fig. S2F).

Monocle decision tree analysis revealed the first differentiation node at the bifurcation of differentiating AMPs/differentiation intermediates into ISC-p and EC lineages (Fig. 2D). We performed gene clustering along these two pseudotemporally ordered branches, identifying five gene groups based on differentiation trajectories and expression timing (fig. S2G and table S4). Groups 4 and 5 comprised genes specifically up-regulated during AMP–to–ISC-p differentiation, with GO analysis linking these genes to ISC fate determination (fig. S2G).

Protein-protein interaction (PPI) enrichment analysis (STRING database) of group 4 genes (table S5) revealed a central network associated with cell fate commitment (cluster 1 in fig. S3). This network featured core modules from the Notch, Wnt, and epidermal growth factor receptor (EGFR) signaling pathways (Fig. 2E). Visualization of ISC markers and pathway-related genes along the AMP-to-ISC-p/EC pseudotemporal axis showed progressive increases in both ISC markers and Notch/Wnt/EGFR pathway components during ISC-p specification (Fig. 2F). This coordinated up-regulation implicates that those three pathways may be involved in the specification of ISC.

### Notch suppression is required for ISC specification

**Fig. 3.**
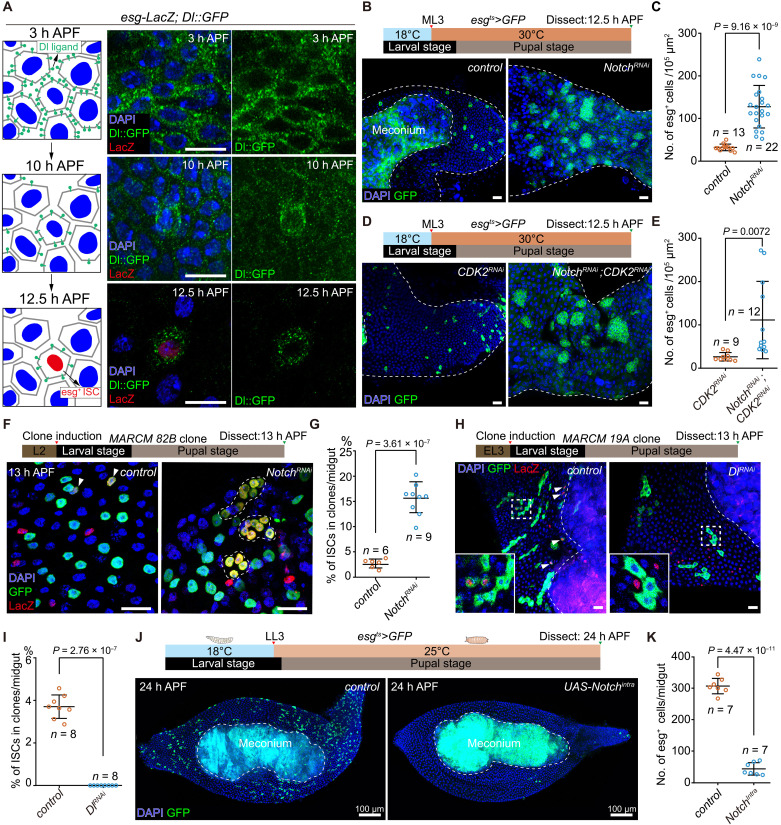
Suppression of Notch signaling is required for ISC specification. (**A**) *Dl*:: GFP gradually reduces from homogeneous expression in AMP cells at 3 hours APF to expression only in *esg*-LacZ^+^(red) ISCs at 12.5 hours APF. Left: Cartoon illustration. Scale bars, 10 μm. (**B** and **C**) Representative images (B) and statistics of ISC number (C) in *control* (*esg^ts^*>*GFP*) and *esg^ts^*>*Notch^RNAi^* midguts at 12.5 hours APF. ISCs were identified as *esg*>GFP^+^ cells. *n*, number of counted images. Each image covers an area of ~10^5^ μm^2^ of the pupal gut. (**D** and **E**) Representative images (D) and statistics of ISC number (E) in *esg^ts^*>*Cdk2^RNAi^* and *esg^ts^*>*Notch^RNAi^; Cdk2^RNAi^* midguts at 12.5 hours APF. *n*, number of counted images. (**F**) L2-induced *control* MARCM clones (nuclear GFP) and *Notch^RNAi^* MARCM clones (nuclear GFP) with the ISC marker *esg*-LacZ staining (red). ISC clone cells in the *control* are indicated by arrowheads, while ISC clusters in *Notch^RNAi^* clones are indicated by dashed circles. (**G**) Statistics of the ISC percentage (%) in MARCM clone cells per midgut. *n*, number of midguts. Clone cells counted: *control*, 5957; and *Notch^RNAi^*, 4497. (**H** and **I**) Representative images (H) and statistics (I) of the ISC percentage (%) showing that ISC cannot be specified from *Dl* knockdown MARCM clone cells. ISC clone cells are indicated by arrowheads. Inset represents close-up views of clones in selected box regions. *n*, number of midguts. Clone cells counted: *control*, 1403; and *Dl^RNAi^*, 374. (**J** and **K**) Representative images (J) and statistics (K) showing the number of ISCs in control and *Notch*-overexpressing midguts at 24 hours APF. *n*, number of midguts. Meconium is outlined by white dashed line in (B), (H), and (J). Data are means ± SD. Statistical analysis by two-tailed unpaired *t* test. Scale bars, 20 μm unless otherwise specified. h, hours.

Given the association between Notch signaling and ISC-p specification, we analyzed the expression pattern of Notch ligand Delta (*Dl*) in early pupal midgut using a *Dl*::green fluorescent protein (GFP) (*49*) reporter. At 3 hours APF, *Dl* was uniformly expressed across the intestinal epithelium (Fig. 3A). By 10 hours APF, *Dl* expression diminished in most epithelial cells, with only scattered cells retaining relatively higher *Dl* levels. At 12.5 hours APF, residual *Dl*-expressing cells exclusively coincided with *esg*-LacZ^+^ cells, confirming their identity as ISCs. Consistent results were obtained using *Dl-Gal4*>*GFP* (fig. S4A) (*50*).

The progressive restriction of *Dl* expression to ISCs suggests that Notch-mediated lateral inhibition governs ISC specification. In this mechanism, *Dl*-expressing cells activate Notch signaling in neighboring cells, suppressing their *Dl* expression (*51*, *52*). This dynamic reduces Notch activation in the original *Dl*^+^ cell, establishing a self-reinforcing loop that amplifies *Dl* expression (*53*, *54*). To test whether Notch suppression promotes ISC specification, we knocked down Notch in AMPs using the temperature-sensitive *esg^ts^* driver from ML3 stage and quantified *esg*^+^ ISCs at 12.5 hours APF. Notch knockdown substantially increased ISC numbers compared to controls, with ISCs forming regional clusters (Fig. 3, B and C), indicating that Notch inhibition enhances ISC specification.

After excluding AMP population changes as a confounding factor (fig. S4, B to D), we investigated whether ISC increase stemmed from enhanced AMP-to-ISC conversion rather than ISC proliferation. Co-knockdown of *Notch* and *Cdk2* (to block cell cycle progression) still produced clustered ISCs at 12.5 hours APF, confirming that Notch inhibition promotes AMP specification into ISCs (Fig. 3, D and E). To further validate this, we generated Notch knockdown MARCM clones in ML3-stage AMP lineages and induced Flp-Out *Notch^RNAi^* clones via *esg^ts^ F/O* (*55*). Both approaches yielded ISC clusters within AMP lineages (Fig. 3, F and G, and fig. S4E), demonstrating that Notch suppression drives ISC specification from AMPs.

Conversely, to test whether Notch activation suppresses ISC specification, we performed two complementary experiments. First, knocking down *Dl* in ML3-induced AMP MARCM clones forced these lineages to rely on Dl from neighboring cells for Notch activation. Second, we expressed a constitutively active form of the Notch receptor (*Notch^intra^*) (*56*) directly within AMP MARCM clones to autonomously activate Notch signaling. In both scenarios, AMP lineages failed to specify ISCs (Fig. 3, H and I; and fig. S4, F to I), demonstrating that Notch activation blocks ISC specification. To further confirm this, we mildly expressed *Notch^intra^* in AMPs starting from LL3 using *esg^ts^* at 25°C. This manipulation resulted in only a negligible number of ISCs being specified throughout the midgut by 24 hours APF, in stark contrast to the robust ISC specification observed in controls (Fig. 3, J and K). Together, these Notch activation experiments clearly demonstrate that suppression of Notch signaling is indispensable for ISC specification.

### Wnt activation is necessary for ISC specification

**Fig. 4.**
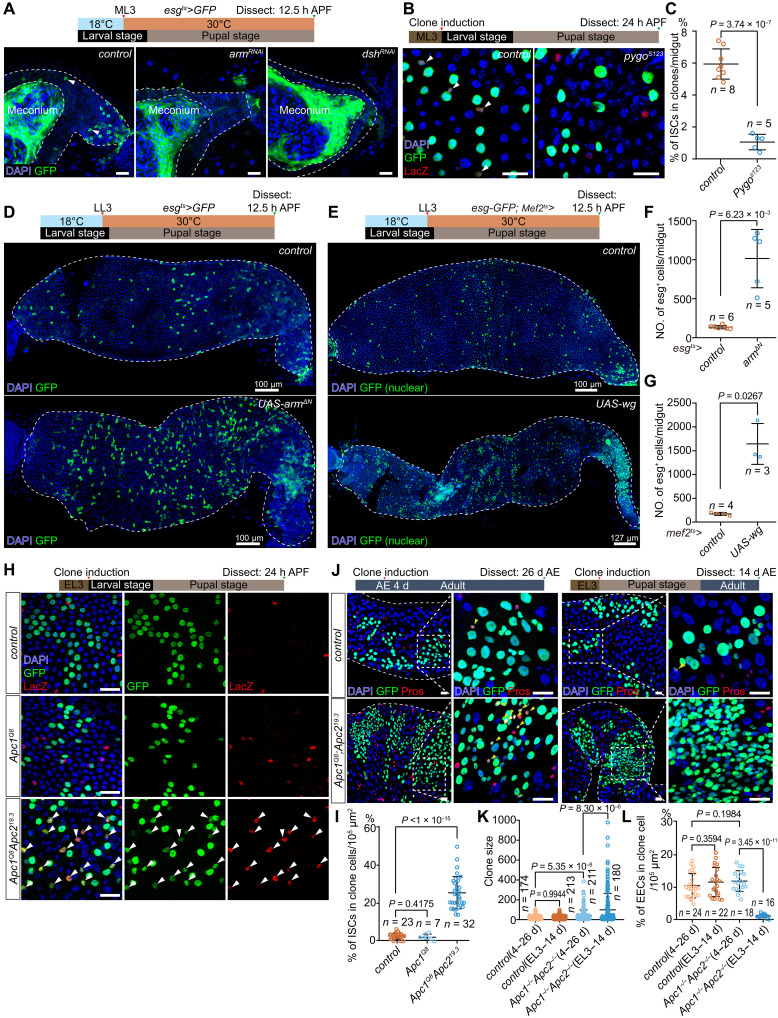
Wnt signaling activation promotes ISC specification. (**A**) Representative images of *control* (*esg^ts^*>*GFP*) and Wnt signaling knockdown (*esg^ts^*>*arm^RNAi^/dsh^RNAi^*) pupal midgut at 12.5 hours APF. *esg*>GFP^+^ ISCs are pointed by white arrowheads. (**B** and **C**) *pygo^S123^* MARCM clones showing substantially lower ISC occurrence in MARCM clone cells than that in *control* clones at 24 hours APF. *n*, number of midguts. Clone cells counted: *control*, 5895; and *pygo^S123^*, 4054. (**D**) Representative images showing a marked increase in the number of ISCs (*esg*>GFP in cytoplasm) in *esg^ts^*>*UAS-arm*^Δ*N*^ pupal midgut compared to *control* (*esg^ts^*>*GFP*) midgut at 12.5 hours APF. (**E**) Representative images showing a marked increase in the number of ISCs (*esg*-GFP in nuclei) in *esg-GFP; Mef2^ts^*>*UAS-wg* pupal midgut compared to *control* (*esg-GFP; Mef2^ts^*>*attp2*) midgut at 12.5 hours APF. (**F** and **G**) Statistics of the ISC number of the midguts in (D) and (E). *n*, number of midguts. (**H**) Representative images of *control*, *Apc1^Q8^*, and *Apc1^Q8^ Apc2^19.3^* MARCM clones (GFP) with ISC marker *esg*-LacZ staining (red, arrowheads) at 24 hours APF. (**I**) Statistics of the ISC percentage in scored MARCM clone cells per image. *n*, number of images. Clone cells counted: *control*, 5895; *Apc1^Q8^*, 2526; and *Apc1^Q8^ Apc2^19.3^*, 6947. (**J**) Representative images of *control* and *Apc1^Q8^ Apc2^19.3^* MARCM clones that (left) induced at 4 days after eclosion (AE) and examined at 26 days AE and (right) induced at EL3 and examined at 14 days AE. Both groups underwent the same tracing time (22 days). Pros staining (red) indicates EEC differentiation (yellow arrowheads). (**K** and **L**) Statistics of clone size (K) and percentage of EECs in scored MARCM clone cells (L) of MARCM clones in clone cells counted: *control* (4 to 26 days), 2971; *control* (EL3 to 14 days), 3634; *Apc1^Q8^ Apc2^19.3^* (4 to 26 days), 8572; and *Apc1^Q8^ Apc2^19.3^* (EL3 to 14 days), 17823. Data are means ± SD. Statistical analysis by two-tailed unpaired *t* test. Scale bars, 20 μm unless otherwise specified. h, hours; d, days.

The incomplete conversion of AMPs into ISCs upon Notch suppression implies that additional mechanisms regulate ISC specification. Given the enrichment of Wnt pathway genes during this process, we blocked Wnt signaling by knocking down two of its core components, *armadillo* (*arm*) (*57*) and *dishevelled* (*dsh*) (*58*, *59*), and overexpressing a dominant-negative form of *pangolin* (*pan*, the *Drosophila TCF*) (*60*) in AMPs starting at the ML3 stage. This resulted in only a few ISCs being specified by 12.5 hours APF (Fig. 4A and fig. S5A). Similarly, *pygo* mutant MARCM clones [targeting the nuclear Wnt machinery (*61*, *62*)] in AMP lineages generated notably fewer ISCs compared to controls at 24 hours APF (Fig. 4, B and C). These findings confirm that Wnt signaling is required for ISC specification.

To determine whether Wnt activation drives ISC specification, we expressed a constitutively active Arm variant (arm^ΔN^) (*63*) in AMPs from LL3 stage. This induced widespread ISC specification across the midgut by 12.5 hours APF (Fig. 4, D and F). Because Wingless (Wg), the Wnt ligand, is secreted by midgut muscle (*60*), we overexpressed Wg using muscle-specific drivers [*vm-Gal4* (*64*) or *Mef2-Gal4* (*65*), fig. S5B]. While Wg overexpression did not alter AMP island numbers or AMP counts (fig. S5, E to G), it robustly increased ISC specification (Fig. 4, E and G; and fig. S5, C and D). Furthermore, activating Wnt signaling via *esg^ts^* F/O clones (>*arm^ΔN^*) enhanced ISC specification within lineages, whereas Wnt inhibition (*>dsh^RNAi^*) reduced ISC numbers (fig. S5H). These experiments collectively demonstrate that Wnt activation promotes AMP-to-ISC specification.

We next analyzed the impact of *Adenomatous polyposis coli* (Apc) mutations, which constitutively activate Wnt signaling (*66*, *67*). EL3-induced *control* (*ry^+^*), *Apc1* single mutant (*Apc1^Q8^*) (*68*), and *Apc1/Apc2* double mutant (*Apc1^Q8^ Apc2^19.3^*) (*69*, *70*) MARCM clones were assessed for *esg-*LacZ^+^ ISC specification at 13 to 15 hours or 24 hours APF. *Apc1^Q8^ Apc2^19.3^* clones exhibited substantially higher ISC specification rates than *controls* or *Apc1^Q8^* clones, indicating that *Apc1/Apc2* loss, but not *Apc1* alone, drives ISC overspecification (Fig. 4, H and I; and fig. S5, I and J). Notably, *Apc1^Q8^ Apc2^19.3^* clones induced during ISC specification formed tightly clustered, hyperproliferative tumor-like lineages lacking Pros^+^ enteroendocrine cell (EEC) differentiation after 22 days (Fig. 4, J to L). In contrast, *Apc1^Q8^ Apc2^19.3^* clones induced 4 days posteclosion (homeostasis stage) generated proliferative cell clusters retaining EEC differentiation. This developmental-stage dependency mirrors the context-specific tumorigenesis observed in mammalian intestinal crypts, where Apc mutations in ISCs, but not differentiated cells, drive tumorigenesis (*71*, *72*).

### EGFR signaling is not required for ISC specification

To evaluate the role of EGFR signaling in ISC specification, we knocked down Ras or Egfr in AMP islands from LL3 stage using *esg^ts^*>*Ras^RNAi^* (*73*) or *Egfr^RNAi^* (*74*). While EGFR signaling knockdown substantially reduced AMP numbers per island (fig. S6, E to G), the number of ISCs specified at 12.5 hours APF remained unchanged relative to controls (fig. S6, A to D). This demonstrates that ISC specification is independent of the initial AMP population size and that EGFR signaling is dispensable for ISC fate acquisition.

Conversely, we generated MARCM clones overexpressing the EGFR effector Raf (*75*, *76*) in EL3-stage AMP lineages. At 13 hours APF, ISC specification rates in Raf-overexpressing clones were comparable to controls (fig. S6, H and I). By 24 hours APF, however, these clones exhibited a ~2-fold increase in ISC numbers (fig. S6, J and K), indicating that EGFR signaling promotes ISC proliferation postspecification but does not influence fate determination.

### ISC specification requires both Notch suppression and Wnt activation

**Fig. 5.**
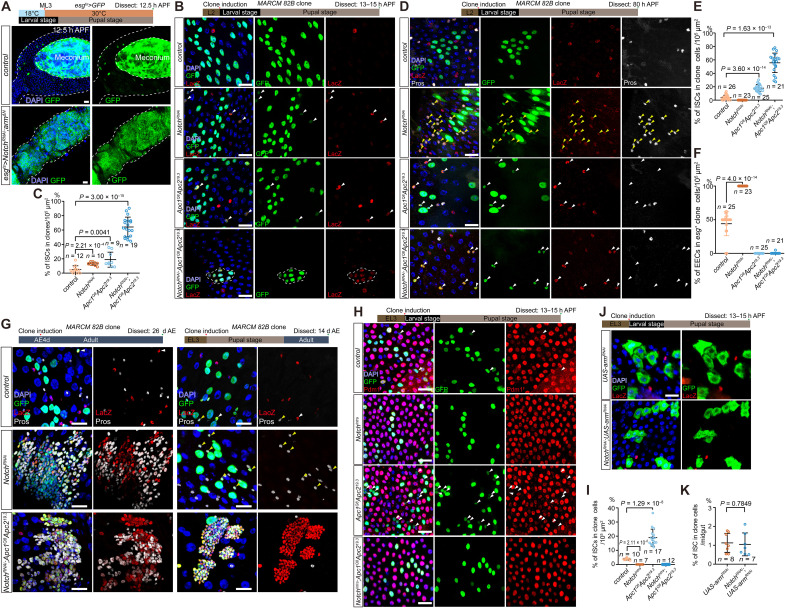
Notch inhibition and Wnt activation cooperatively drive ISC specification. (**A**) Representative images of *esg*>GFP (green) labeled ISCs in *control* (*esg^ts^*>*GFP*) and *esg^ts^*>*Notch^RNAi^* + *arm^ΔN^* midguts at 12.5 hours APF. (**B**) Representative images of indicated MARCM clones (GFP) with ISC marker *esg*-LacZ staining (red) at 13 to 15 hours APF. Arrowheads and dashed line indicate ISC clone cells. (**C**) Statistics of the ISC percentage in indicated MARCM clone cells per image. *n*, number of images. Clone cells counted: *control*, 1089; *Notch^RNAi^*, 589; *Apc1^Q8^ Apc2^19.3^*, 733; and *Notch*^*RNA*i^ + *Apc1^Q8^ Apc2^19.3^*, 1116. (**D** to **F**) Representative images (D), statistics of the ISC (*esg*-LacZ^+^) percentage in clone cells (E), and the statistics of the EEC (Pros^+^, yellow arrowheads) percentage in *esg*-LacZ^+^ clone cells (F) of indicated MARCM clones at 80 hours APF. *n*, number of images (10^5^ μm). Clone cells counted: *control*, 2508; *Notch^RNAi^*, 3081; *Apc1^Q8^ Apc2^19.3^*, 1756; and *Notch^RNAi^* + *Apc1^Q8^ Apc2^19.3^*, 910. (**G**) Representative images of indicated MARCM clones that induced at 4 days AE and examined at 26 days AE (left) or induced at EL3 and examined at 14 days AE (right). Both groups underwent the same tracing time (22 days). White arrowheads indicate *esg*-LacZ^+^ clone cells, and yellow arrowheads indicate Pros^+^ EEC clone cells. (**H** and **I**) Representative images (H) and statistics of the ISC (GFP^+^Pdm1^−^, arrowheads) percentage (I) of indicated MARCM clone cells at 13 to 15 hours APF. *n*, number of images. Clone cells counted: *control*, 3261; *Notch^intra^*, 608; *Apc1^Q8^ Apc2^19.3^*, 2201; and *Notch^intra^* + *Apc1^Q8^ Apc2^19.3^*, 906. (**J** and **K**) Representative images (F) and statistics of the ISC (*esg*-LacZ, red) percentage (G) of indicated MARCM clone cells at 13 to 15 hours APF. *n*, number of midguts. Clone cells counted: *UAS-arm^RNAi^*, 1874; and *Notch^RNAi^* + *UAS-arm^RNAi^*, 2651. Data are means ± SD. Statistical analysis by two-tailed unpaired *t* test. Scale bars, 20 μm.

To determine whether simultaneous Notch suppression and Wnt activation suffice to convert AMPs into ISCs, we combined Notch knockdown (*esg^ts^*>*Notch^RNAi^*) with Wnt activation (*>arm*^Δ*N*^) in ML3-stage AMPs. By 12.5 hours APF, all AMPs became *esg*>GFP^+^ ISCs, demonstrating that dual Notch suppression and Wnt activation fully specify AMP-to-ISC fate (Fig. 5A).

To further validate this, we generated EL3-stage MARCM clones with Notch knockdown (Notch^RNAi^) and Wnt activation via *Apc1/Apc2* double mutations, arm^ΔN^ overexpression, or Wg expression. At 13 to 15 hours APF, clones with combined Notch suppression and Wnt activation showed substantially higher ISC specification rates than either perturbation alone, with some clones fully converting to *esg*^+^ ISCs (Fig. 5, B and C; and fig. S7, A to C). Therefore, our lineage tracing data demonstrate that AMP can be specified as an ISC under dual Notch inhibition and Wnt activation.

Notably, because Notch activation is required to inhibit EEC differentiation following pupal ISC asymmetric division (*24*), ISCs specified by Notch knockdown alone differentiated entirely into Pros^+^ EECs by 80 hours APF (Fig. 5, D to F). In contrast, ISCs specified by Wnt activation alone or dual Notch suppression/Wnt activation locked ISCs in an undifferentiated state (Fig. 5, D to F; and fig. S7, D to F). Notably, Notch^RNAi^ + *Apc1^Q8^ Apc2^19.3^* clones induced during ISC specification formed undifferentiated, hyperproliferative ISC-like tumors at 14 days after eclosion (AE) (Fig. 5G). In contrast, clones induced at 4 days AE generated tumors retaining EEC differentiation. Together, these results suggest that sustained inhibition of Notch and activation of Wnt during the ISC specification stage converts AMP into undifferentiated, continuously proliferating ISCs.

To define the genetic interplay between Notch and Wnt, we activated Wnt (Apc1/Apc2 mutant clones) while overexpressing Notch^intra^. Despite Wnt activation, Notch^intra^ overexpression forced all clone cells to differentiate into Pdm1^+^ ECs, phenocopying Notch activation alone (Fig. 5, H and I). Conversely, dual Notch inhibition and Wnt suppression failed to enhance ISC specification beyond Wnt suppression alone (Fig. 5, J and K). These epistasis experiments confirm that ISC specification necessitates both Notch suppression and Wnt activation, with these pathways functioning interdependently.

To investigate whether Wnt signaling directly regulates Dl expression, we introduced the *Dl::GFP* reporter into backgrounds with either inhibited (*UAS-pan^DN^*) or hyperactivated (*UAS-arm*^Δ*N*^) Wnt signaling, under the control of the *esg^ts^* driver. When Wnt signaling was inhibited from the ML3 stage onward, although RFP^+^ ISCs failed to be specified by 12.5 hours APF, scattered epithelial cells still retained *Dl::GFP* expression (fig. S8), indicating that Wnt suppression does not abolish Dl expression. Conversely, constitutive activation of Wnt signaling via *arm^ΔN^* did not lead to noticeable up-regulation of *Dl::GFP* in the epithelium, including within RFP^+^ ISCs (fig. S8). These results suggest that Wnt signaling does not directly activate Dl transcription but rather establishes a permissive spatial niche within which Notch-mediated lateral inhibition can subsequently operate to specify ISCs.

### Notch-mediated lateral inhibition specifies ISC from Wnt*-*activated zones

**Fig. 6.**
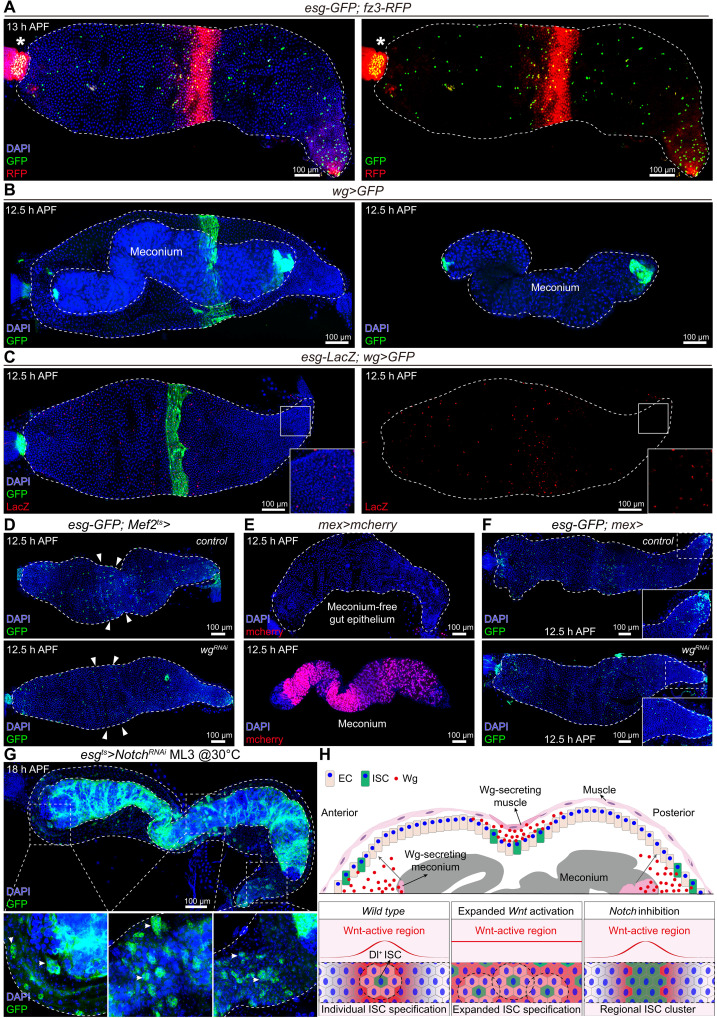
Notch specifies ISC from Wnt activation zones via lateral inhibition. (**A**) Representative image of *fz3*-RFP and *esg*-GFP staining in the midgut at 13 hours APF. Meconium was removed from the midgut except in (B) and (G). White dashed line outlines the midgut. Asterisk (*) indicates the cardia. (**B**) Representative image of *wg*>GFP expression in the midgut at 12.5 hours APF. Left: Midgut with meconium. Right: Meconium only. (**C**) Representative image of the *esg*-LacZ (red) and *wg*>GFP (green) in the midgut at 12.5 hours APF. Inset represents close-up views in selected box regions. We increased the brightness and contrast of the *esg*-lacZ signal to make it visible. (**D**) Representative images of *esg*-GFP expression in *control* (*esg-GFP; Mef2^ts^*>*attp2*) and muscle ligand knockdown (*esg-GFP; Mef2^ts^*>*UAS-wg^RNAi^*) midguts at 12.5 hours APF. Arrowheads indicate endogenous *wg* expression domains. (**E**) Representative images of *mex*>mCherry expression in the midgut epithelium (top) or meconium (bottom) at 12.5 hours APF. Dashed line show the outline of the midguts. (**F**) Representative images of *esg*-GFP expression in *control* (*esg-GFP; mex*>*attp2*) and meconium ligand knockdown (*esg-GFP; mex*>*UAS-wg^RNAi^*) midguts at 12.5 hours APF. Inset represents close-up views in selected box regions. (**G**) Representative images of *esg*>GFP expression in *esg^ts^*>*Notch^RNAi^* midguts at 18 hours APF. Inset represents close-up views in selected box regions. (**H**) ISC specification model: (top) Wg secretion from muscle and meconium establishes ISC-specification zones. Notch mediates lateral inhibition within the zones to refine ISC selection. Bottom: ISC distribution patterns under wild-type, Wnt overexpression, and Notch-inhibited conditions. Data are presented as means ± SD. All images are representative of ≥20 biological replicates. Scale bars, 100 μm. h, hours.

To investigate how Notch and Wnt signaling coordinate to specify ~150 ISCs from ~6000 AMPs, we mapped Wnt activation patterns. Using *pygo* mutant MARCM clones, we observed that *frizzled3*-RFP (*fz3*-RFP), a Wnt signaling reporter (*77*, *78*), was absent in pupal midgut clone cells (fig. S9A), validating its utility for monitoring Wnt activation. At 13 hours APF, *fz3*-RFP exhibited strong circular expression in the midgut’s middle epithelium region and localized to anterior/posterior epithelial domains (Fig. 6A), indicating spatially restricted Wnt activation.

Correlating with Wnt-active regions, using a Gal4 knock-in at the Wg locus (*79*), *wg{KO; Gal4}*>*GFP* revealed ligand expression in the middle circular muscle of the midgut and the anterior/posterior ends of the meconium epithelium (Fig. 6B). As the meconium epithelium originates from larval midgut ECs (*11*, *13*, *80*), we confirmed *wg{KO; Gal4}*>*GFP* expression at larval LL3 stage in anterior/posterior ECs (fig. S9B). During pupal meconium formation, these Wg-expressing domains remained closely adjacent to the midgut epithelium until 10 hours APF before retracting centrally (fig. S9C), suggesting meconium-derived Wnt ligand patterns anterior/posterior regions.

Consistent with Wnt’s spatial activation, ISCs emerged in the midgut’s middle domain (*22*) and radiated from anterior/posterior ends toward the middle by 12.5 hours APF (Fig. 6, A and C, and also Figs. 1C and 4, D and E; and figs. S5C and S6, A and C). To test muscle-derived Wg’s role in ISC specification, we knocked down Wg using a muscle-specific driver (*Mef2-Gal4*). This selectively reduced middle ISC numbers without affecting anterior/posterior ISC densities (Fig. 6D and fig. S9D), implicating muscle-derived Wg in middle specification. To assess the role of meconium-derived Wg in ISC specification, we knocked down Wg using *mex-Gal4* (*81*, *82*), which is expressed in all larval midgut ECs (fig. S8E). However, *mex-Gal4* activity was absent in the anterior-end meconium epithelium, restricting Wg knockdown to posterior meconium domains (Fig. 6E and fig. S9E). Consequently, *mex-Gal4*–driven Wg knockdown selectively reduced posterior ISC numbers at 12.5 hours APF (Fig. 6F and fig. S9F), suggesting that meconium-derived Wg is required for ISC specification.

We hypothesized that Notch-mediated lateral inhibition specifies ISCs in Wnt-active anterior/middle/posterior domains, achieving a selection ratio of ~1/40 (150/6000 AMPs). In support of this, Notch knockdown resulted in restricted ISC clustering within Wnt-active domains (Fig. 6G). Together, these data support a two-step selection model: Wnt activation defines spatial niches permissive for ISC specification, while Notch-mediated lateral inhibition selects individual *Dl*^+^ ISCs within these niches (Fig. 6H).

### Notch suppression promotes mouse Lgr5-EGFP^+^ ISC-p specification

**Fig. 7.**
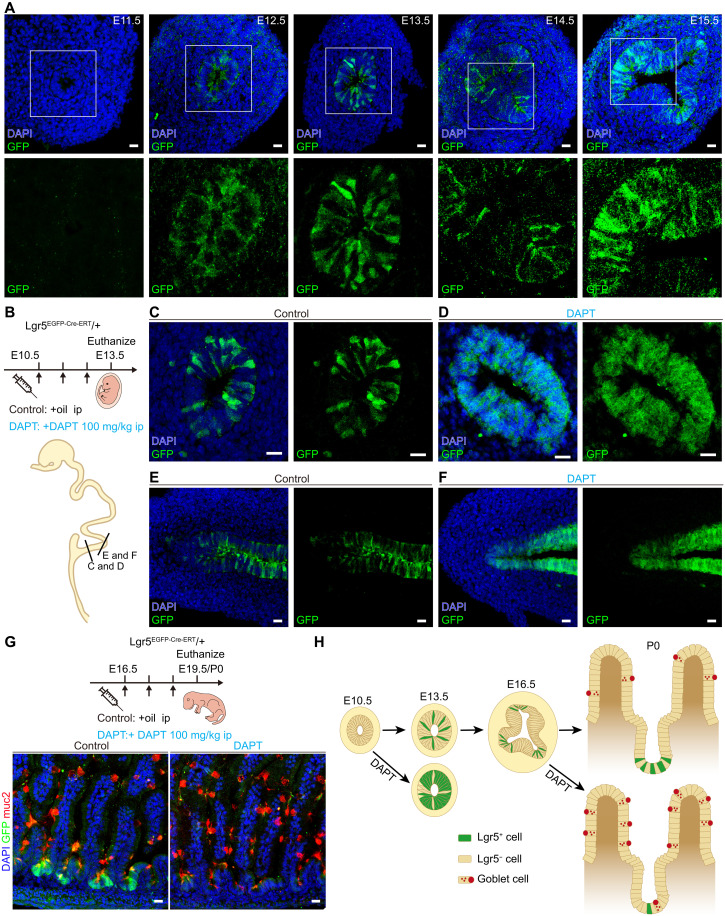
Notch suppression promotes Lgr5-EGFP^+^ progenitor specification in the mouse embryonic intestine. (**A**) Cross sections of gastrointestinal tract from *Lgr5^EGFP-Cre-ERT^* embryos showing the distribution of Lgr5^+^ cells at E11.5 to E15.5. The enlarged image below illustrates the gradual emergence of Lgr5-EGFP^+^ cells during embryonic intestinal development (*n* = 15 slices per mouse, three mice analyzed). (**B**) Experimental schematic for control and DAPT injection from E10.5 to E12.5. The schematic drawing below shows the embryonic gastrointestinal tract at E13.5. Black lines show the section levels for corresponding images (C) to (F). (**C** to **F**) Cross sections [(C) and (D)] and longitudinal sections [(E) and (F)] showing Lgr5-EGFP staining of control and DAPT-treated mice in E13.5 gastrointestinal tract (*n* = 15 slices per mouse, three mice analyzed). (**G**) Top: Experimental schematic for control and DAPT injection from 16.5 to E18.5. Bottom: Representative images of Lgr5-EGFP and Muc2 staining (red) in the P0 small intestines of control and DAPT-treated mice (*n* = 25 slices per mouse, three mice analyzed). ip, intraperitoneal. (**H**) Notch signaling switches from inhibiting ISC specification to maintaining ISC stemness during embryonic gut development. Scale bars, 20 μm. All images are representative of ≥3 biological replicates.

During mouse embryogenesis, Wnt signaling activation drives Lgr5^+^ ISC-p specification (*17*), consistent with our findings in *Drosophila* ISC specification. Through daily observation of embryonic intestines in *Lgr5^EGFP-Cre-ERT^* mice, we detected sparse Lgr5-EGFP^+^ progenitors at E12.5, followed by a patterned, spaced distribution at E13.5 (Fig. 7A). By E14.5 to E15.5, Lgr5-EGFP^+^ progenitor clusters localized to intervillus regions, where crypt folding initiated (*83*). The spaced emergence of Lgr5-EGFP^+^ progenitors at stage E13.5 parallels the distribution of *Drosophila* ISCs resulting from Notch-mediated lateral inhibition, indicating that Notch may similarly regulate Lgr5^+^ progenitor specification in the mouse embryonic intestinal epithelium.

To test this, we intraperitoneally injected pregnant mice with the γ-secretase inhibitor DAPT (to block Notch signaling) (*84*) at E10.5, before Lgr5-EGFP^+^ progenitor emergence. Compared to corn oil–injected controls, DAPT-treated E13.5 embryos exhibited contiguous Lgr5-EGFP^+^ domains rather than spaced progenitors (Fig. 7, B to F), demonstrating that Notch suppression expands Lgr5-EGFP^+^ progenitor specification during embryonic development.

Notably, Notch activation exhibits stage-specific roles: While it restricts ISC specification during embryogenesis, it is essential for maintaining adult ISCs (*84*, *85*). To assess this functional shift, we administered DAPT at E16.5 (post–crypt formation). In P0 intestines, Lgr5-EGFP^+^ cells were markedly reduced, accompanied by an increased goblet cell population (Fig. 7G), phenocopying Notch inhibition in adult mice (*84*). This reveals a developmental switch in Notch function: It restricts ISC specification during E10.5 to E13.5 but transitions to maintaining ISC stemness and blocking secretory differentiation after crypt formation (Fig. 7H).

## DISCUSSION

On the basis of our findings, we propose a revised model for ISC specification during *Drosophila* pupal metamorphosis. In contrast to the previous hypothesis that each larval AMP island preselects one ISC, our data demonstrate that ISCs are specified from a broad epithelial pool through the coordinated action of spatially restricted Wnt signaling and Notch-mediated lateral inhibition (fig. S10). Specifically, Wnt ligands secreted from circular muscles and meconium define discrete “competent zones” along the anterior-posterior axis of the early pupal gut. Within these domains, Notch lateral inhibition enables the emergence of Dl-high cells, which subsequently adopt ISC fate (fig. S10). This mechanism ensures the specification of ~150 ISCs from nearly 6000 AMPs by 12.5 hours APF.

Although the theory that each AMP island in the larval midgut provides an adult ISC is appealing (*10*), our study reveals that the number of ISCs specified in the early pupal midgut is substantially lower than the AMP island count. Intriguingly, EGFR pathway knockdown reduces AMP cell numbers but does not substantially alter ISC quantity, indicating a robust developmental mechanism independent of specification pool size. Assuming constant Notch lateral inhibition patterning, Wnt activation range appears to determine ISC numbers. Given that AMP island size/number in the larval stage can be altered by bacterial infection or injury (*86*), this AMP-independent specification mechanism facilitates intestinal homeostasis resetting.

Our previous work showed that Notch activation in *Drosophila* pupal ISCs following asymmetric divisions maintains stem cell fate and prevents EEC differentiation (*24*, *26*), mirroring Notch’s role in mammalian Lgr5^+^ ISCs for maintaining stem cell fate and preventing goblet cell differentiation (*84*, *85*, *87*). This study further reveals that transient Notch suppression through lateral inhibition is essential for specifying ISC fate from equipotent intestinal epithelium in both *Drosophila* and mouse gut development, creating a spaced ISC distribution pattern analogous to the *Drosophila* neuroblast specification (*88*). This evolutionary convergence suggests Notch functions as a stage-dependent molecular switch: Initial inhibition enables progenitor specification while subsequent activation maintains stem cell identity. This paradigm explains why Notch inhibition promotes ISC formation during early human embryonic gut development but causes ISC loss at later stages (*89*), indicating conserved temporal switching.

While Wnt activation maintains Lgr5^+^ ISCs in mammals and Apc mutations cause undifferentiated adenomas (*71*, *90*, *91*), Wnt pathway knockdown does not notably affect ISC numbers in adult *Drosophila* midguts (*60*, *78*, *92*, *93*). Furthermore, Apc mutant–driven ISC proliferation in adult flies does not impair differentiation (*60*, *70*, *78*, *93*, *94*), raising questions about Wnt pathway conservation. However, our data show that Wnt signaling inhibition during ISC specification markedly reduces ISC numbers, while Apc mutation-induced hyperactivation during this window produces undifferentiated tumors. Thus, Wnt signaling conservation in *Drosophila* occurs during ISC specification of early pupal midgut development.

In mammalian crypt-villi structures, crypt base ISCs receive Wnt ligands from subepithelial telocytes (*95*) and Notch ligands from secretory progenitor cells (*96*), activating respective signaling pathways to maintain stem cell fate (*3*). In contrast, the adult *Drosophila* midgut lacks crypt-villi architecture (*97*), does not require Wnt for ISC maintenance (*60*, *70*, *78*, *93*, *94*), and uses bidirectional Notch signaling between ISCs and daughters to determine EC/EEC differentiation (*24*). Direct comparisons between *Drosophila* adult midgut and mammalian crypt-villi structures obscure evolutionary conservatism. However, temporal analysis reveals that the *Drosophila* pupal midgut parallels crypt base development (requiring Wnt/Notch for stemness), while adult midgut resembles the transient-amplifying region of the mammalian small intestine (Notch-dependent differentiation without Wnt requirement) (*98*, *99*). This temporal-spatial correspondence establishes the *Drosophila* pupal midgut as a conserved model for studying Notch/Wnt-driven ISC specification in mammalian development and carcinogenesis.

## MATERIALS AND METHODS

### *Drosophila* husbandry

Standard cornmeal fly food was prepared using the following recipe: 210 g of dry-inactivated yeast (Angel Yeast Co., Ltd.), 900 g of yellow cornmeal, 120 g of soy flour, 100 g of agar (Solarbio, catalog no. A8190), 800 ml of light corn syrup, 0.3 g of levofloxacin (Dalian Meilun), 15 g of methyl 4-hydroxybenzoate (Biosharp), and 12 liters of water. Flies were maintained at 25°C and 65% relative humidity on a 12-hour light/dark cycle, unless otherwise indicated. Female animals were used in all experiments. The fly lines used in this study are listed in data S1.

### Immunostaining and fluorescent microscopy

Midguts from fly pupae or larvae were dissected in 1× phosphate-buffered saline (PBS; Solarbio, catalog no. P1010). Samples were fixed in 4% formaldehyde (Sigma-Aldrich, catalog no. F8775) for 2 to 3 hours at room temperature (RT), followed by three 20-minwashes in 0.3% PBT (1× PBS containing 0.3% Triton X-100; Sangon Biotech, catalog no. A110694-0500), except for anti-Mew antibody staining, in which case samples were fixed in 4% formaldehyde for 20 min.

Midguts were incubated with primary antibodies for 3 hours at RT under gentle shaking at the following dilutions: chicken anti-GFP (1:10,000; Abcam, catalog no. ab13970), rabbit anti-GFP (1:10,000; Abcam, catalog no. 6556); mouse anti-Prospero (1:200; Developmental Studies Hybridoma Bank, catalog no. 528440), rabbit anti-PH3 (1:10,000; Millipore, catalog no. MMI-06-570), chicken anti-LacZ (1:10,000; Abcam, catalog no. ab9361), rabbit anti-Pdm1 (1:200; gift from X. Yang’s lab, Zhejiang University), and mouse anti-Mew (1:100; Developmental Studies Hybridoma Bank, catalog no. AB_528303, a gift from R. Xi’s lab, Tsinghua University) (*37*). After primary antibody incubation, samples were washed three times with 1× PBS for 20 min at RT under shaking and then incubated with secondary antibodies for 3 hours at RT. Unbound secondary antibodies were removed by three 20-min washes in 0.3% PBT. Nuclei were counterstained with 100 μl of 4′,6-diamidino-2-phenylindole (DAPI; 0.1 μg/ml; Sigma-Aldrich, catalog no. D9542) for 5 min, followed by three 5-min washes in 0.3% PBT. Samples were mounted in 70% glycerol (Sinopharm Chemical Reagent, catalog no. 10010618) and imaged using a Carl Zeiss LSM 800 confocal microscope equipped with Plan-Apochromat 20×/0.8 M27 and 63×/1.40 Oil DIC M27 objectives. Immersol 518 F (Zeiss) was used as the imaging medium. All images were acquired at 25°C using ZEN 2.1 software and processed for merging and resizing in Adobe Photoshop and Adobe Illustrator.

Special notes: (i) Meconium removal: For pupal intestines, meconium was carefully removed postfixation by puncturing the tissue with fine forceps before proceeding with staining. (ii) *Dl::*GFP signal stability: *Dl::*GFP fluorescence exhibited rapid signal decay, requiring imaging within 2 hours after staining completion.

### Dissecting *Drosophila* at specific time points

LL3 larval staging: Female LL3 larvae exhibiting wandering behavior (climbing the vial walls) were transferred to fresh cornmeal food vials and designated as the LL3 stage.

Pupariation onset: Newly formed white pupae were collected into new vials to establish the 0 hours APF reference time point. All vials were maintained at 25°C.

Dissection schedule: Pupae were dissected and processed for immunofluorescence at the following APF stages: 7, 12.5, 16, 20, 24, 28, 32, 36, 40, and 48 hours APF.

### Knockdown of *Cdk2* during early pupal development

*esg^ts^*>*GFP* (*control*) and *esg^ts^*>*UAS-Cdk2^RNAi^* flies were maintained at 18°C under standard conditions. Newly formed white pupae (0 hours APF) were collected into new vials. At 0 hours APF, samples were shifted to 30°C to induce Gal4-dependent gene expression. Pupae were dissected and processed for immunofluorescence at 12.5 and 24 hours APF.

### Elimination of ISCs during early pupal development

Our preliminary results suggest that ISCs were specified at 12.5 hours APF. To target these newly generated ISCs, we used conditional cell ablation using *esg^ts^*>*UAS-hid*. Flies were maintained at 18°C until pupal development. At 5 hours APF, the *esg-GFP esg^ts^*>*UAS-hid* pupae were transferred to 30°C to activate *hid* expression, followed by a return to 18°C at 12.5 hours APF to terminate the ablation. Midguts were subsequently dissected and analyzed by immunofluorescence staining at 24 hours APF.

### MARCM clonal induction in larval AMPs across developmental stages

To generate MARCM clones at specific stages of AMP development, we crossed *MARCM19A* and *esg-LacZ; FRT19A; esg-LacZ/CyO* (1:3 male-to-female ratio) or *MARCM82B* and *yw122; esg-LacZ; FRT82B ry/SM6^TM6B* flies. Adult flies were transferred to an embryo collection chamber overnight. Embryo-containing agar plates were collected the following morning and incubated at 25°C. First-instar larvae (L1) were picked out from plates 10 hours postcollection to establish a developmental time point. Subsequently, 40 to 50 healthy larvae were transferred to fresh cornmeal-based food vials and staged as L1. Developmental stages were tracked daily (L2, 1 day post-L1; EL3, 2 days post-L1; ML3, 3 days post-L1; and LL3, 4 days post-L1).

For clone induction, larvae at defined stages (L1 to LL3) were subjected to heat shock at 37°C for 1 hour. Larvae raised under optimal nutritional conditions typically pupariated by day 5. Dissections were performed at designated time to analyze AMP lineages.

### Sample preparation for snRNA-seq

*Drosophila w^1118^* flies were maintained at 25°C. White female pupae were staged as 0 hours APF and transferred to new vials. At 8 hours APF, pupal midguts were dissected by three researchers in parallel within a 4-hour window to collect ~250 midguts per replicate. Dissections were performed in ice-cold ribonuclease-free 1 × PBS (Leagene, catalog no. NH0012; Ca^2+^/Mg^2+^-free) to isolate the midgut epithelium. The hindgut, Malpighian tubules, and meconium were carefully removed. Dissected epithelia were pooled into low-adhesion EP tubes (Eppendorf, catalog no. 022431021) prechilled on dry ice. Residual PBS introduced during tissue transfer was later aspirated. This protocol was repeated across four independent biological replicates to obtain 1000 midguts total. Tubes were snap frozen in liquid nitrogen for 10 min and stored at −80°C.

Frozen midgut epithelia from four replicates were thawed on ice, combined into a single tube, and centrifuged at 10,000*g* for 2 min at 4°C. Supernatant was carefully removed, and the pellet was refrozen in liquid nitrogen for 3 min. Tissue was homogenized in 3 ml of lysis buffer using a dounce homogenizer (10 strokes with a loose pestle, 5 strokes with a tight pestle). Lysates were incubated for 5 min at 4°C, diluted with 5 ml of wash buffer, and filtered through a 30-μm cell strainer. Nuclei were pelleted at 500*g* for 5 min, washed three times in wash buffer, and resuspended by gentle pipetting (8 to 10 cycles). For purity, nuclei were layered onto a 29% OptiPrep density cushion (Sigma-Aldrich) and centrifuged at 10,000*g* for 30 min. Purified nuclei were washed three times in wash buffer and resuspended in 1 ml of buffer.

Approximately 140,000 nuclei (94.2% single-nucleus purity) were processed using the 10x Genomics Chromium Single Cell 3′ Kit v3.1. Nuclei and GemCode gel beads were coencapsulated into droplets using the Chromium Controller. Reverse transcription, cDNA amplification, and library construction followed the manufacturer’s protocol. Libraries were sequenced on an Illumina NovaSeq 6000 system (Novogene) with paired-end 150–base pair reads.

### Bioinformatics analysis of single-cell nuclei

Raw snRNA-seq reads were aligned to the *Drosophila melanogaster* reference genome (BDGP6.32, Ensembl Metazoa release 109) using CellRanger v5.0.0 (10x Genomics). Initial quality control and preprocessing were performed with Seurat V5.0.3. Cells expressing fewer than 400 or more than 2000 genes, or with mitochondrial RNA content exceeding 5%, were excluded. Ribosomal genes were removed from downstream analyses. Potential doublets were identified and filtered using DoubletFinder v2.0.6 with default parameters and an expected doublet rate of 0.75%. After filtering, 5384 high-quality nuclei were retained. Gene expression matrices were normalized using the LogNormalize method in Seurat. Principal components analysis was performed on the top 2000 variable genes, and the first 30 principal components were used for *t*-SNE dimensionality reduction. Cluster resolution parameters (0.01 to 2.0) were systematically tested, with a final resolution of 0.9 selected to identify 16 transcriptionally distinct clusters. Cluster identities were assigned by cross-referencing marker genes from published single-cell atlases of the adult midgut (*31*, *39*), canonical lineage-specific markers (*31*), and literature-curated gene signatures (*10*, *100*, *101*).

Trajectory analysis of seven intestinal epithelial cell clusters was conducted using Monocle2 v2.30.1. Highly variable genes identified by Monocle2 were selected as ordering genes. Dimensionality reduction was performed with the DDRTree algorithm, and pseudotemporal ordering was inferred on the basis of gene expression dynamics. Branch-dependent gene regulation was analyzed using Monocle2’s BEAM (Branched Expression Analysis Modeling) tool. GO enrichment analysis of trajectory-associated modules was performed with Metascape V3.5.2. PPI networks for genes enriched in ISC-p1/p2 specification were reconstructed using the STRING database (minimum interaction score, 0.7). Networks were partitioned into 26 functional communities via *k*-means clustering and visualized in Cytoscape v3.10.3.

### Temperature-sensitive *Gal4* is induced at the ML3 stage

Eggs from fly crosses were cultured at 18°C for 9 days. To synchronize knockdown initiation at the ML3 larval stage, progeny was transferred to 30°C for 24 hours, activating the tissue-specific Gal4 drivers, and, then, female larvae adhering to the inner walls of culture tubes were collected.

A portion of the larvae were immediately dissected for analysis as needed for a particular experiment. The remaining larvae were maintained at 30°C until white puparium formation (designated as 0 hours APF). Pupae were maintained at 30°C and subsequently dissected at 12.5 hours APF developmental time points.

### *esg^ts^ F/O* clone analysis from ML3

*esg-LacZ; UAS-GFP*, *esg-LacZ; UAS-Notch^RNAi^*, *esg-LacZ; UAS-arm*^Δ*N*^, and *esg-LacZ; UAS-dsh^RNAi^* flies were crossed with *esg^ts^ F/O* and then cultured at 18°C incubator for 9 days; both groups of larvae were transferred to a 30°C environment for 24 hours. Then, female larvae adhering to the inner walls of culture tubes were collected. The larvae were maintained at 30°C until white puparium formation (designated as 0 hours APF). Pupae were maintained at 30°C and dissected at 24 hours APF and stained for immunofluorescence.

### Visualization of intestinal muscle expression at 12.5 hours APF

*Mef2-Gal4* and *vm-Gal4* driver lines were crossed with *UAS-GFP* at 25°C to generate muscle-specific GFP expression. LL3 female larvae were transferred to fresh cornmeal-based food vials. Pupariation was monitored hourly, and newly formed white pupae were collected, staged as 0 hours APF. Pupae were dissected at 12.5 hours APF and stained for immunofluorescence.

### Knockdown of *wg* in muscles from EL3

Eggs from *esg-GFP; Mef2^ts^*>*attp2* (control) and *esg-GFP; Mef2^ts^*>*UAS-wg^RNAi^* crosses were cultured for 7 days at 18°C, both groups of larvae were transferred to 30°C to activate Gal4 function for 48 hours. Then, female larvae adhering to the inner walls of culture tubes were collected. The larvae were maintained at 30°C until white puparium formation (0 hours APF). Pupae were maintained at 30°C and dissected at 12.5 hours APF and stained for immunofluorescence.

### Knockdown of *wg* in meconium during early pupal stage

*esg-GFP; mex*>*attp2* (*control*) and *esg-GFP; mex*>*UAS-wg^RNAi^* were cultured at 25°C, and LL3 female larvae were placed in new culture tubes. The larvae were maintained until white puparium formation (designated as 0 hours APF). Pupae were dissected at 12.5 hours APF and stained for immunofluorescence.

### MARCM clones are induced at EL3 or 4 days AE in *Drosophila* development

Eggs from crosses of *esg-LacZ; FRT82B ry Notch*^*RNAi*^ MARCM, *esg-LacZ; FRT82B ry APC1*^*Q8*^ *APC2*^*19.3*^ MARCM, *esg-LacZ; FRT82B ry Notch*^*RNAi*^; *APC1*^*Q8*^ *APC2*^*19.3*^ MARCM, and *esg-LacZ; FRT82B ry* MARCM flies were collected and reared at 25°C. A subset of eggs was allowed to develop to 4 days AE. Flies were heat shocked at 37°C for 1 hour, returned to 25°C (hs@AE 4d), and dissected at 26 days AE for analysis. Another subset was heat shocked at 37°C for 1 hour at EL3, returned to 25°C (hs@EL3), and allowed to develop to adulthood. These flies were dissected at 14 days AE.

### Mouse models and housing

Adult female C57BL/6 wild-type mice were obtained from Vital River Laboratory Animal Technology Co., Ltd. (Beijing, China). *Lgr5^EGFP-IRES-CreERT2^* mice were acquired from Shanghai Model Organisms Center Inc. (NM-KI-200154). All procedures were approved by the Institutional Animal Care and Use Committee (IACUC) of Huazhong University of Science and Technology (Wuhan, China) and performed in strict accordance with the guidelines for the care and use of laboratory animals at the Experimental Animal Center of Huazhong University of Science and Technology (Wuhan, China) (IACUC approval no. 2024-4782). Animals were maintained under a 12-hour light-dark cycle (lights on at 8:00 a.m.) with controlled environmental conditions: ambient temperature 21° ± 2°C and humidity 50 ± 5%. For timed pregnancies, proestrous females were housed with fertile males overnight, and embryonic age was determined by vaginal plug detection. The day of plug observation was designated as E0.5.

To inhibit Notch signaling, pregnant mice received intraperitoneal injections of DAPT (γ-secretase inhibitor, MedChemExpress, catalog no. 208255-80-5, 100 mg/kg per day dissolved in corn oil) for 3 consecutive days starting at E10.5 or E16.5. Vehicle control groups received equivalent volumes of corn oil administered on the same schedule. All dams were euthanized by cervical dislocation 24 hours after the final injection for subsequent analyses.

### Intestinal cryosection preparation and immunofluorescence

Embryonic intestines were harvested at specified developmental stages, fixed in 4% paraformaldehyde (BioSharp, catalog no. BL539A) for 12 hours at 4°C, and dehydrated through a sucrose gradient (20 to 30% in PBS; Sinopharm Chemical, catalog no. 10021418). Tissues were then embedded in optimal cutting temperature compound (Yeasen, catalog no. 36309ES61) and cryosectioned at 20-μm thickness in both transverse and longitudinal orientations.

For immunofluorescence, sections were rehydrated in PBS containing 0.1% Triton X-100 (PBST) for 30 min, blocked with 5% normal goat serum (Boster Bio, catalog no. AR1009) for 1 hour at RT, and incubated with primary antibodies diluted in blocking solution overnight at 4°C. The following antibodies were used: chicken anti-GFP (1:300; Abcam, catalog no. ab13970) and rabbit anti-Muc2 (1:200; ABclonal, catalog no. A14659). After three 8-min PBST washes, sections were incubated with species-matched Alexa Fluor–conjugated secondary antibodies (1:500; Invitrogen) for 1 hour at RT. Nuclear counterstaining was performed with DAPI (1:10,000; Sigma-Aldrich, catalog no. D9542) for 10 min, followed by three 5-min washes in 0.3% PBST. Slides were mounted with 70% glycerol/PBS mounting medium (National Pharmaceutical, catalog no. 10010618) and imaged using a Zeiss LSM 800 confocal microscope. Image processing was performed with Adobe Photoshop CC and Illustrator CS6 (Adobe Systems).

### Statistical analysis

Statistical analysis was performed using Prism 6 (GraphPad Software). Two-tailed unpaired Student’s *t* test and one-way analysis of variance (ANOVA) with Bonferroni’s multiple-comparisons test were performed to assess differences. All statistics results are presented as means ± SD. All of the statistical details of the experiments can be found in the figures and figure legends.
