# Supplementary material for: Drosophila and mouse intestinal stem cells are spatiotemporally specified by Notch suppression and Wnt activation
Source: Sci Adv. 2025 Dec 3;11(49):eady7272. doi: 10.1126/sciadv.ady7272 (PMC12674121; doi:10.1126/sciadv.ady7272)
Supplement: Supplementary file 1 — Figs. S1 to S10 Data S1 Legends for tables S1 to S5 [file sciadv.ady7272_sm.pdf]

Science Advances
AAAS

Supplementary Materials for

Drosophila and mouse intestinal stem cells are spatiotemporally specified by Notch suppression and Wnt activation

You Wu *et al.*

Corresponding author: Rong Lin, linrong@hust.edu.cn; Zheng Guo, guozheng@hust.edu.cn

Sci. Adv. **11**, eady7272 (2025)
DOI: 10.1126/sciadv.ady7272

The PDF file includes:

Figs. S1 to S10
Data S1
Legends for tables S1 to S5

Other Supplementary Material for this manuscript includes the following:

Tables S1 to S5

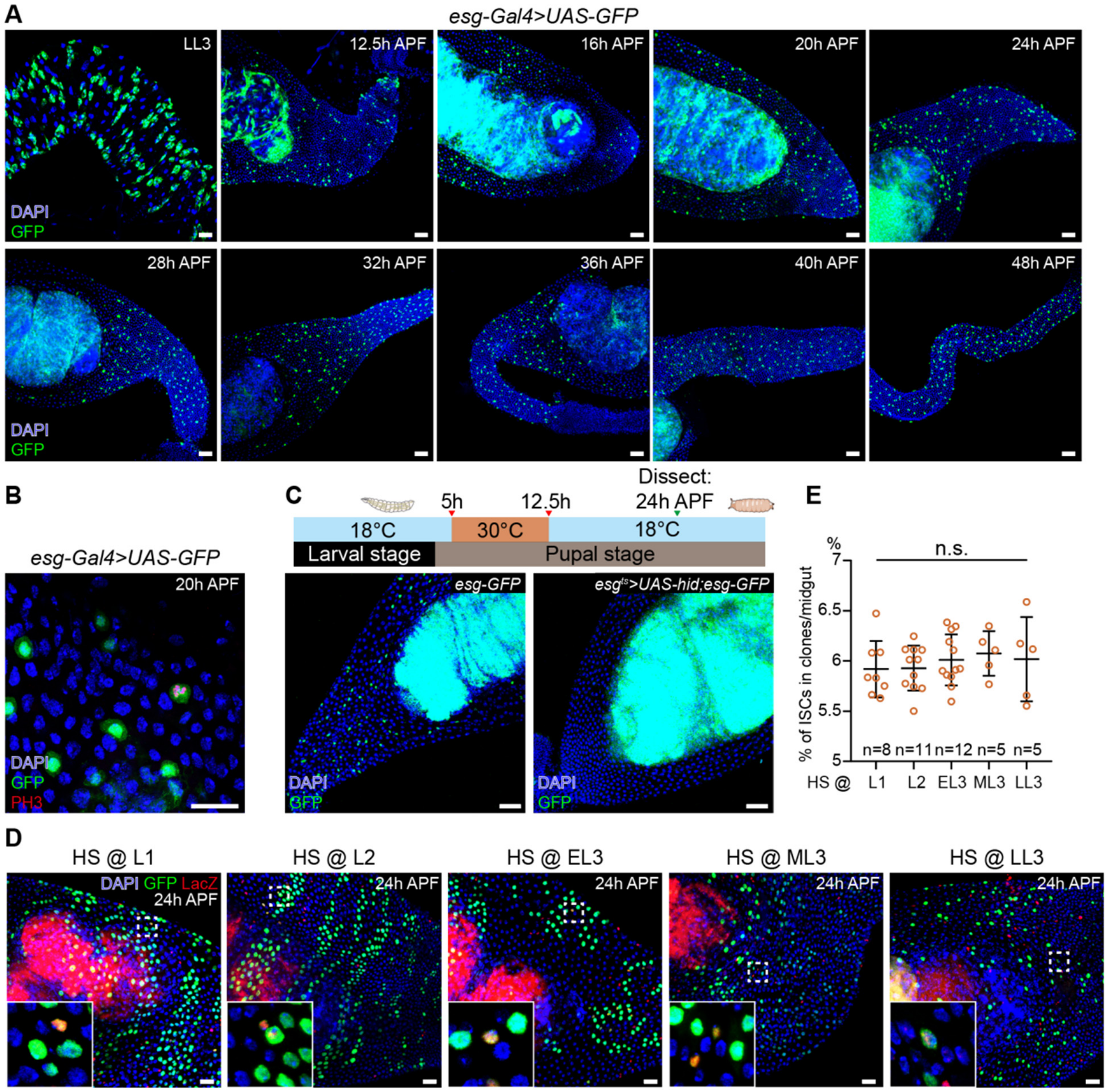

Fig. S1. ISCs has been specified in the pupal midgut epithelium at 12.5h APF.
(A) Representative images of *esg*$^+$ (*esg>GFP*) cell expression in the *Drosophila* intestinal epithelium at indicated developmental time points. **(B)** *esg>GFP* pupal midgut stained with phospho-histone H3 (PH3, red) at 20h APF. **(C)** Upper: Schematic of the genetic manipulations. Lower: Representative images of *esg*$^+$ (*esg*-GFP) ISCs in control and *esg*^{ts}*>UAS-hid* midguts at 24h APF. **(D)** Representative images of nuclear-GFP labeled MARCM clones in 24h APF pupal midguts induced at indicated developmental stages. *esg*-LacZ$^+$ (red) staining was used to show the ISCs. Inset represents close-up views of the *esg*-LacZ$^+$ clone cells in selected box regions. **(E)** Statistics of the ISC percentage (%) in MARCM clone cells per midgut. n, number of pupal midguts. ns, $p>0.05$ by one-way ANOVA. Clone cells counted in these midguts: L1 10153, L2 14998, EL3 18059, ML3 8633, and LL3 3309. Data are mean ± SD. n, number of midguts. Statistical analysis by one-way ANOVA with Bonferroni's multiple-comparisons test. Scale bars, 20 μm.

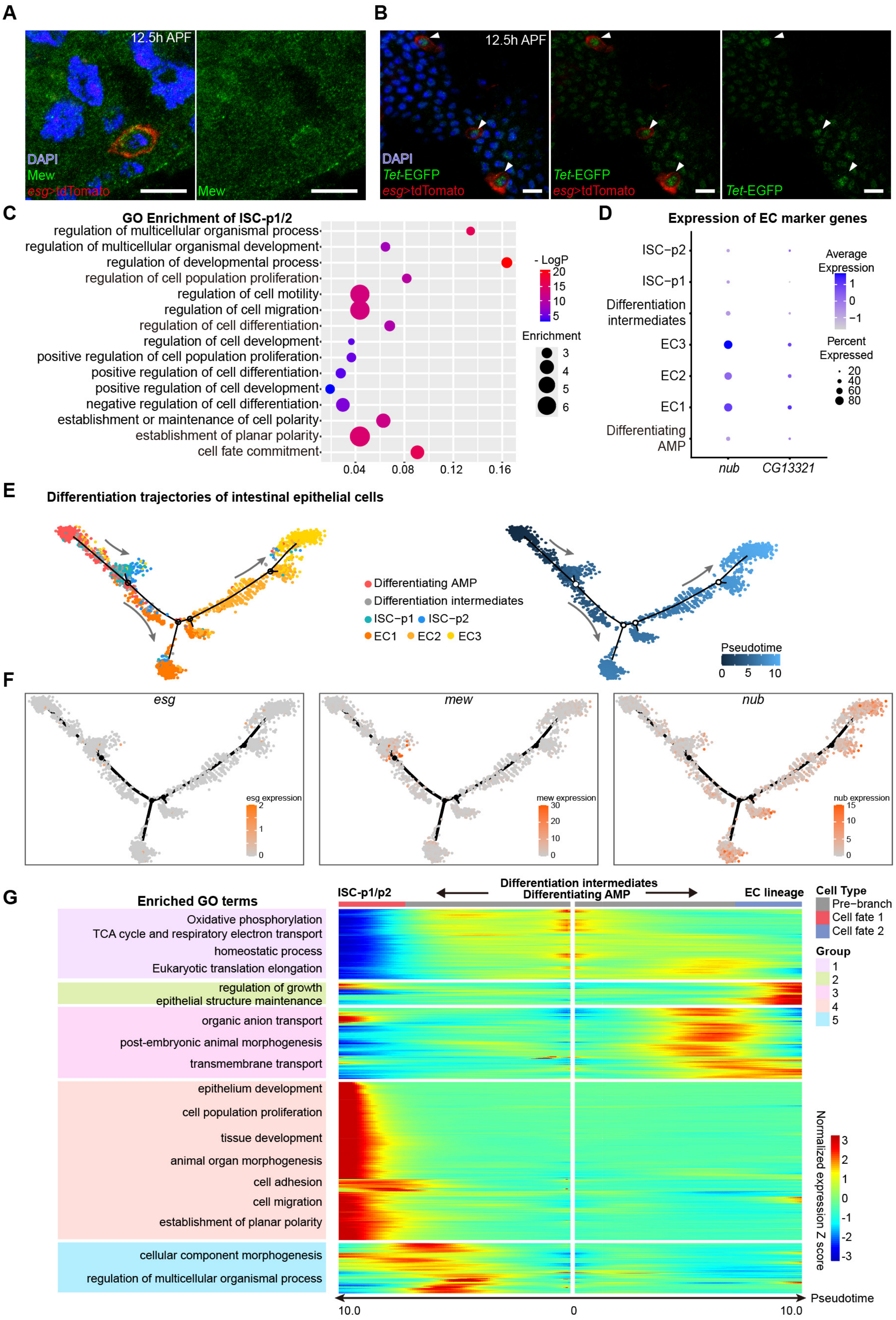

A
12.5h APF
DAPI
Mew
esg>tdTomato
Mew
B
12.5h APF
DAPI
Tet-EGFP
esg>tdTomato
Tet-EGFP
esg>tdTomato
Tet-EGFP
C
GO Enrichment of ISC-p1/2
regulation of multicellular organismal process
regulation of multicellular organismal development
regulation of developmental process
regulation of cell population proliferation
regulation of cell motility
regulation of cell migration
regulation of cell differentiation
regulation of cell development
positive regulation of cell population proliferation
positive regulation of cell differentiation
positive regulation of cell development
negative regulation of cell differentiation
establishment or maintenance of cell polarity
establishment of planar polarity
cell fate commitment
0.04 0.08 0.12 0.16
- LogP
20 15 10 5
Enrichment
3 4 5 6
D
Expression of EC marker genes
ISC−p2
ISC−p1
Differentiation intermediates
EC3
EC2
EC1
Differentiating AMP
nub
CG13321
Average Expression
1 0 −1
Percent Expressed
20 40 60 80
E
Differentiation trajectories of intestinal epithelial cells
Differentiating AMP
Differentiation intermediates
ISC−p1
ISC−p2
EC1
EC2
EC3
Pseudotime
0 5 10
F
esg
esg expression
2 1 0
mew
mew expression
30 20 10 0
nub
nub expression
15 10 5 0
G
Enriched GO terms
Oxidative phosphorylation
TCA cycle and respiratory electron transport
homeostatic process
Eukaryotic translation elongation
regulation of growth
epithelial structure maintenance
organic anion transport
post-embryonic animal morphogenesis
transmembrane transport
epithelium development
cell population proliferation
tissue development
animal organ morphogenesis
cell adhesion
cell migration
establishment of planar polarity
cellular component morphogenesis
regulation of multicellular organismal process
ISC-p1/p2
Differentiation intermediates
Differentiating AMP
EC lineage
Cell Type
Pre−branch
Cell fate 1
Cell fate 2
Group
1
2
3
4
5
Normalized expression Z score
3 2 1 0 -1 -2 -3
Pseudotime
10.0
0
10.0

Fig. S2. Gene expression clustering at the fate branch point from Differentiating AMP to ISC-p1/2 and ECs.

(A) Co-localization of Mew (green) and esg>tdTomato (red) in the pupal gut at 12.5h APF. **(B)** Representative images of Tet-EGFP expression (green) in *esg>tdTomato* pupal midgut at 12.5h APF. **(C)** Gene Ontology (GO) enrichment analysis of clusters 5 and 6 (annotated as ISC-p1 and p2). **(D)** Dotplot of EC marker expression (*nub* [*Pdm1*], *CG13321*) across clusters. Dot size corresponds to the percentage of expressing cells; color intensity reflects mean expression. **(E)** Pseudotime trajectory analysis of midgut cells. Left: Trajectory colored by annotated cell types. Right: Pseudotemporal ordering (pale colors = later pseudotime). Arrow shows the direction of the cell fate decision. **(F)** The expression dynamics of esg, nub, and mew genes along the pseudotime trajectory, colored by gene expression level (orange: high expression; gray: low expression). **(G)** GO analysis of modules created by clustering the two main branches from the lineage tree in a pseudo-temporal order. Cell fate commitment is reflected in this analysis. The middle represents the start pseudo-time of AMP differentiating. From this point, one lineage moves to the ISC-p1/2 and the other moves to the EC. Scale bars, 10 µm.

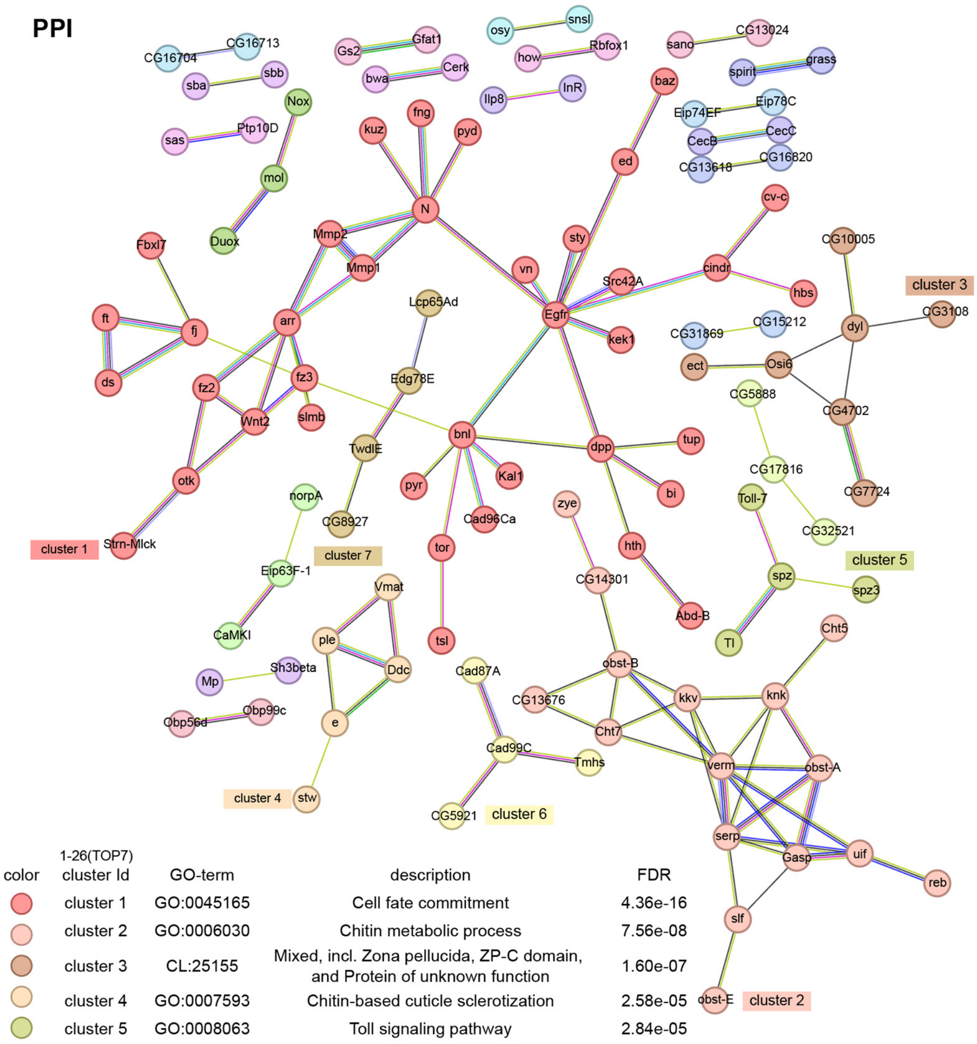

color	1-26(TOP7) cluster Id	GO-term	description	FDR
	cluster 1	GO:0045165	Cell fate commitment	4.36e-16
	cluster 2	GO:0006030	Chitin metabolic process	7.56e-08
	cluster 3	CL:25155	Mixed, incl. Zona pellucida, ZP-C domain, and Protein of unknown function	1.60e-07
	cluster 4	GO:0007593	Chitin-based cuticle sclerotization	2.58e-05
	cluster 5	GO:0008063	Toll signaling pathway	2.84e-05
	cluster 6	GO:0044331	Cell-cell adhesion mediated by cadherin	0.0174
	cluster 7	GO:0005214	Structural constituent of chitin-based cuticle	3.19e-05

Fig. S3. The PPI enrichment analysis of the cluster 4 in Figure S2D.
26 cluster were identified by the STRING database. The top 7 clusters were analyzed by GO enrichment.

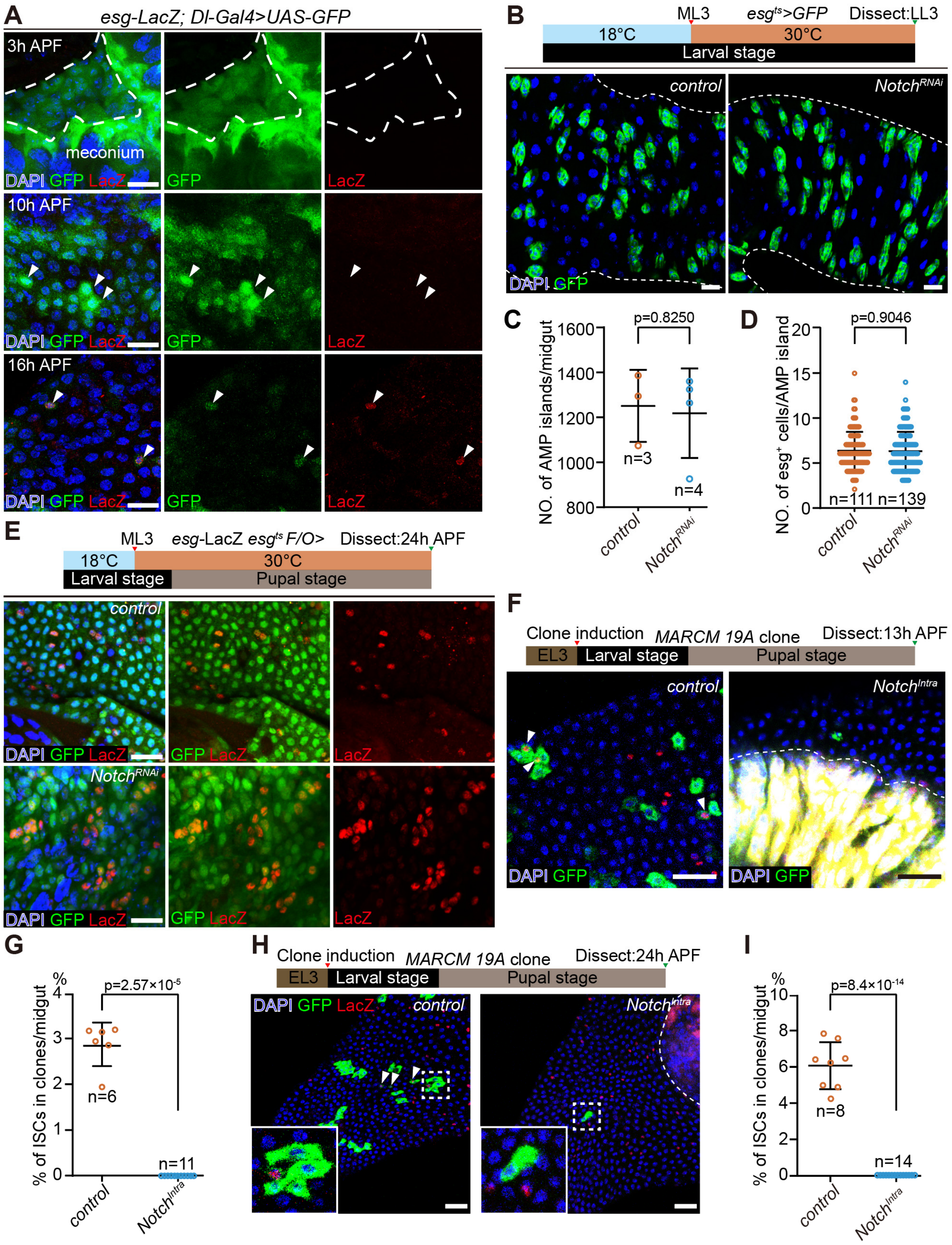

Fig. S4. The ISC specification requires suppression of Notch signaling.
(A) GFP driven by *Dl*-Gal4 gradually reduces from homogeneous expression in AMP cells at 3h APF to expression only in *esg*-LacZ^{+}(red) ISCs at 12.5h APF. Dashline shows the outline of the intestinal epithelial region, and arrowheads indicate GFP-enriched cells. **(B)** Representative images of AMP cells in *control* (esg^{ts}>*GFP*) and esg^{ts}>$Notch^{RNAi}$ at the LL3 stage. Dashline show the outline of the larval gut. **(C and D)** Statistics of AMP islands (C) per gut and AMP cells (D) per AMP island in *control* (esg^{ts}>*GFP*) and Notch signaling inhibited LL3 midguts. n, number of midguts (C) or number of AMP islands (D). **(E)** Representative images of *esg*-LacZ (red) staining in *control* (*esg-LacZ;* esg^{ts} *F/O>GFP*) and *esg-LacZ;* esg^{ts} *F/O>*$Notch^{RNAi}$ midguts at 24h APF. Notch knockdown and lineage tracing was induced at ML3. Lineage cells were labeled by GFP. **(F and G)** Representative images (F) and statistics of the ISC percentage (G) showing that ISC cannot be specified from Notch activated MARCM clone cells at 13h APF. ISCs in MARCM clones are indicated by arrowheads. n, number of midguts. Clone cells counted: *control* 981, and $Notch^{Intra}$ 39. **(H and I)** Representative images (H) and statistics of the ISC percentage (I) showing that ISC cannot be specified from Notch activated MARCM clone cells at 24h APF. ISCs in MARCM clones are indicated by arrowheads. Inset represents close-up views of clones in selected box regions. n, number of midguts. Clone cells counted: *control* 1487, and $Notch^{Intra}$ 60. Meconium is outlined by white dashline in F and H. Data are mean ± SD. Statistical analysis by two-tailed unpaired t-test. Scale bars, 20 μm.

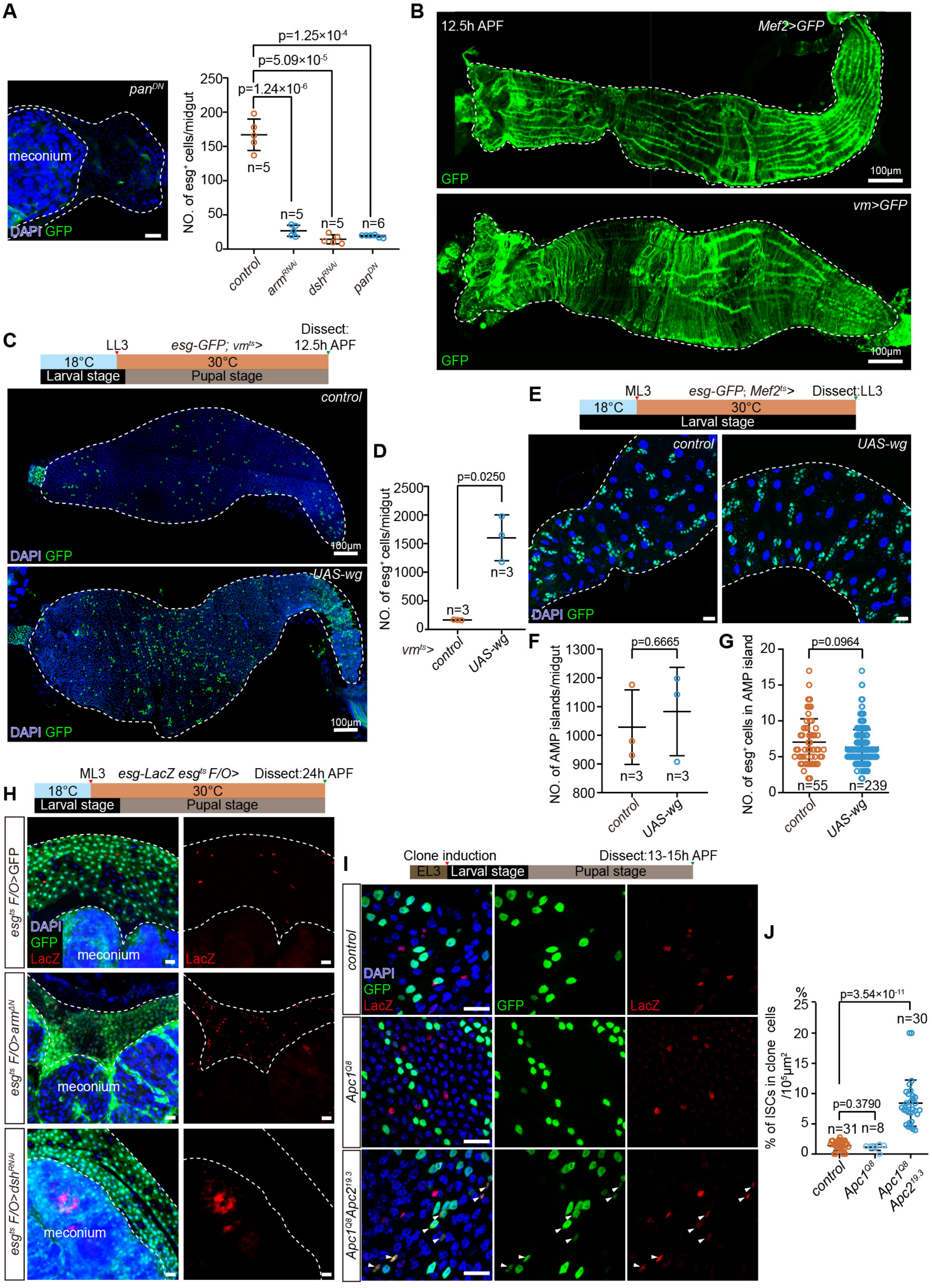
A
panDN
meconium
DAPI GFP
p=1.25×10-4
p=5.09×10-5
p=1.24×10-6
NO. of esg+ cells/midgut
n=5
n=5
n=5
n=6
control
armRNAi
dshRNAi
panDN
B
12.5h APF
Mef2>GFP
GFP
100μm
vm>GFP
C
LL3
esg-GFP; vmts>
Dissect: 12.5h APF
18°C
30°C
Larval stage
Pupal stage
control
UAS-wg
DAPI GFP
D
p=0.0250
NO. of esg+ cells/midgut
n=3
n=3
vmts>
control
UAS-wg
E
ML3
esg-GFP; Mef2ts>
Dissect:LL3
F
p=0.6665
NO. of AMP islands/midgut
G
p=0.0964
NO. of esg+ cells in AMP island
n=55
n=239
H
ML3
esg-LacZ esgts F/O>
Dissect:24h APF
esgts F/O>GFP
esgts F/O>armΔN
esgts F/O>dshRNAi
DAPI
GFP
LacZ
meconium
I
Clone induction
EL3
Dissect:13-15h APF
control
Apc1Q8
Apc1Q8Apc219.3
J
p=3.54×10-11
p=0.3790
% of ISCs in clone cells /105μm2
n=31
n=8
n=30

Fig. S5. Wnt signaling activation is not only necessary for but also promotes ISC specification.

(A) Representative image of ISCs overexpressing the dominant-negative form of *pan* (the *Drosophila TCF*) in the pupal midgut at 12.5 h APF (left), and quantitative of ISCs in control (esg^{ts}>*GFP*) and experimental (esg^{ts}>arm^{RNAi}/dsh^{RNAi}/pan^{DN}) midguts at the same stage (right). n, number of midguts. **(B)** Midgut muscle-driver *Mef2-Gal4* (top) and *vm-Gal4* (Bottom) drives GFP expression in the midgut at 12.5h APF. Dashline show the outline of the midguts. **(C and D)** Representative images (C) and statistics (D) showing a dramatic increase in the number of ISCs (*esg*-GFP in nuclei) in *esg-GFP;* vm^{ts}>*UAS-wg* pupal midgut compared to *control* (*esg-GFP;* vm^{ts}>*attp2*) midgut at 12.5h APF. n, number of midguts. **(E)** Representative images of AMP cells in *control* (*esg-GFP;* $Mef2^{ts}$>*attp2*) and *esg-GFP;* $Mef2^{ts}$>*UAS-wg* at the LL3 stage. Dashline show the outline of the larval gut. **(F and G)** Statistics of AMP islands (F) per gut and AMP cells (G) per AMP island in *control* (*esg-GFP;* $Mef2^{ts}$>*attp2*) and Wnt signaling activated (*esg-GFP;* $Mef2^{ts}$>*UAS-wg*) LL3 midgut. n, number of midguts (F) or number of AMP islands (G). **(H)** Representative images of ISCs (*esg*-LacZ, red) staining in *control* (*esg-LacZ;* esg^{ts} *F/O>GFP*), *esg-LacZ;* esg^{ts} *F/O*>$arm^{\Delta N}$, and esg^{ts} *F/O*>dsh^{RNAi} midguts at 24h APF. Dashed lines show the outline of the pupal intestine. Wnt signaling manipulations and lineage tracing was induced at ML3. Lineage cells were labeled by GFP. **(I)** Representative images of *control*, $Apc1^{Q8}$, and $Apc1^{Q8}$ $Apc2^{19.3}$ MARCM clones (GFP) with ISC marker *esg*-LacZ staining (red, arrowheads) dissected at 13-15h APF. **(J)** Statistics of the ISC percentage in MARCM clone cells per image. n, number of images. Clone cells counted: *control* 8224, $Apc1^{Q8}$ 1860, $Apc1^{Q8}$ $Apc2^{19.3}$ 3910.

The midgut and meconium boundary is outlined by white dashline in (A) and (H). Data are mean ± SD. Statistical analysis by two-tailed unpaired t-test. Scale bars, 20 µm unless otherwise specified.

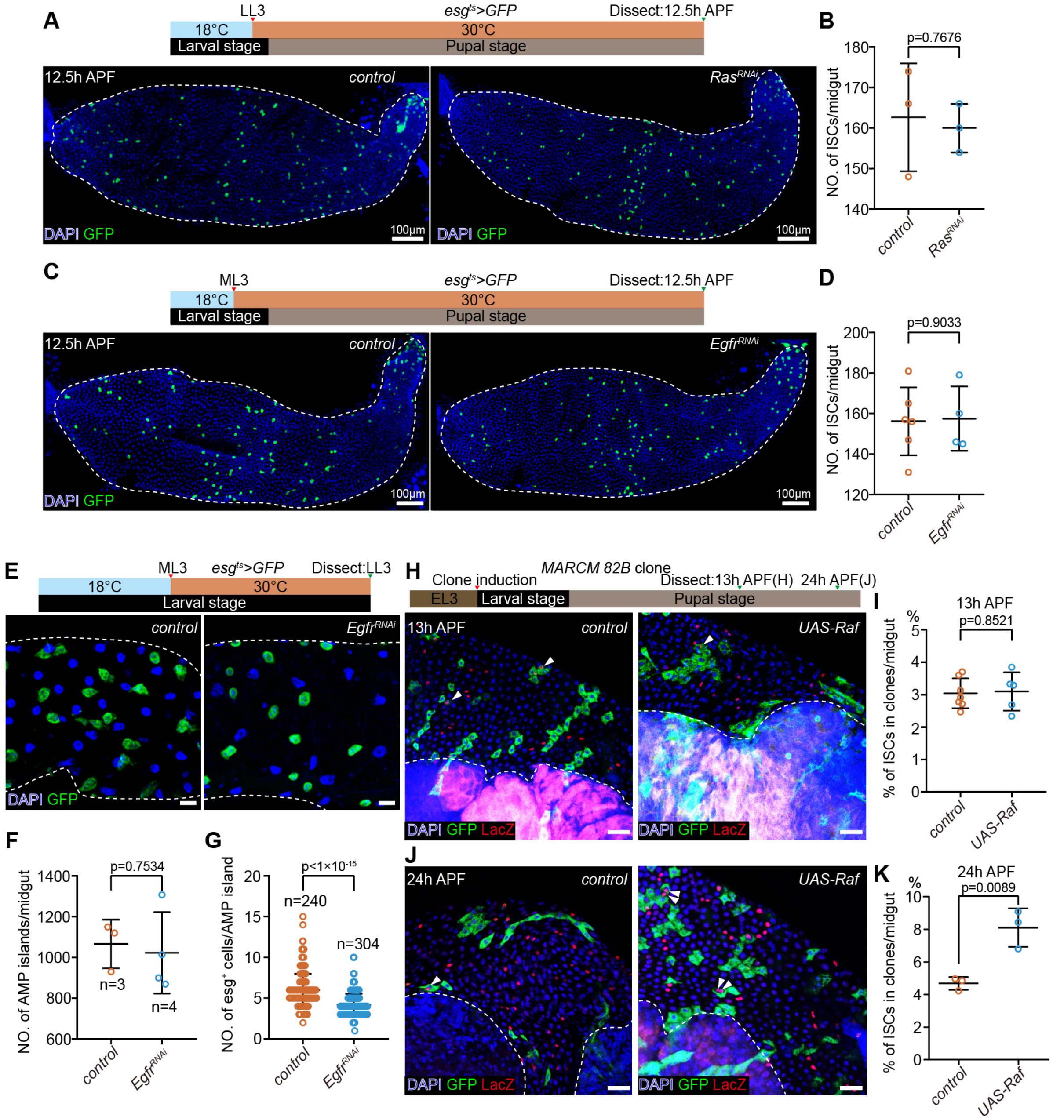

Fig. S6. EGFR signaling is not required for ISC specification.

(A and B) Representative images (A) and statistics of ISC number (B) in *control* (*esg*^{ts}>*GFP*) and *esg*^{ts}>*UAS-Ras*^{RNAi} midguts at 12.5h APF. n, number of midguts. **(C and D)** Representative images (C) and statistics of ISC number (D) in *control* (*esg*^{ts}>*GFP*) and *esg*^{ts}>*UAS-Egfr*^{RNAi} midguts at 12.5h APF. n, number of midguts. **(E)** Representative images of AMP cells in *control* (*esg*^{ts}>*GFP*) and *esg*^{ts}>*UAS-Egfr*^{RNAi} at the LL3 stage. Dashline show the outline of the larval gut. **(F and G)** Statistics of AMP islands (F) per gut and AMP cells (G) per AMP island in *control* (*esg*^{ts}>*GFP*) and EGFR signaling inhibited (*esg*^{ts}>*UAS-Egfr*^{RNAi}) LL3 midgut. n, number of midguts (F) or number of AMP islands (G). **(H and I)** Representative images (H) and statistics (I) of the ISC (*esg*-LacZ^{+}, red, arrowheads) percentage (%) showing that *UAS-Raf* MARCM clones did not

specify more ISCs at 13h APF. n, number of midguts. Clone cells counted: *control* 1823, *UAS-Raf* 3895. **(J and K)** Representative images (J) and statistics of the ISC (*esg*-LacZ$^+$) percentage (K) of *control* and *UAS-Raf* MARCM clones at 24h APF. n, number of midguts. Clone cells counted: *control* 549, *UAS-Raf* 1096. Data are mean ± SD. Statistical analysis by two-tailed unpaired t-test. Scale bars, 20 µm unless otherwise specified.

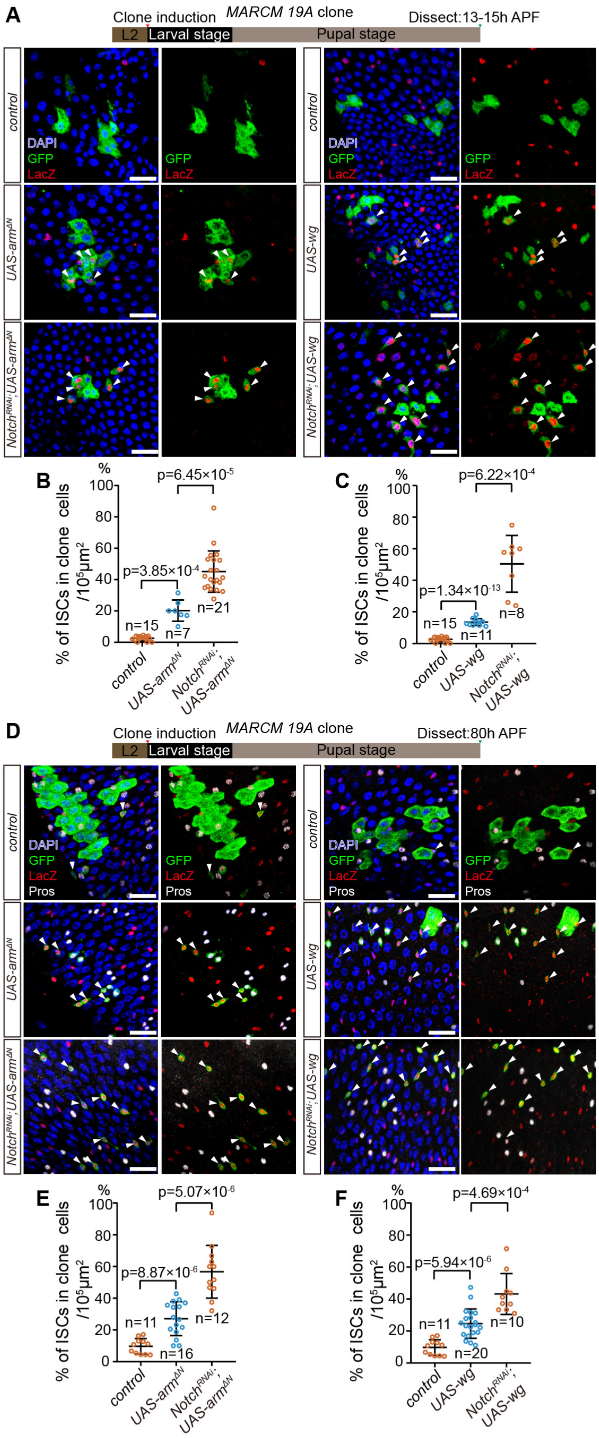

Fig. S7. ISC specification requires both Notch suppression and Wnt activation.
(A-C) Representative images (A) and statistics of the ISC (*esg*-LacZ$^+$) percentage (B and C) of indicated MARCM clone cells at 13-15h APF. n, number of images. Clone cells counted: *control* 551, *UAS-arm*$^{\Delta N}$ 172, *Notch*^{RNAi}+*UAS-arm*$^{\Delta N}$ 1512, *UAS-wg* 662, *Notch*^{RNAi}+*UAS-wg* 163. **(D-F)** Representative images (D) and statistics of the ISC (*esg*-LacZ$^+$) percentage (E and F) of indicated MARCM clone cells at 80h APF. n, number of images. Clone cells counted: *control* 1598, *UAS-arm*$^{\Delta N}$ 837, *Notch*^{RNAi}+*UAS-arm*$^{\Delta N}$ 347, *UAS-wg* 1178, *Notch*^{RNAi}+*UAS-wg* 220. Data are mean ± SD. Statistical analysis by two-tailed unpaired t-test. Scale bars, 20 μm.

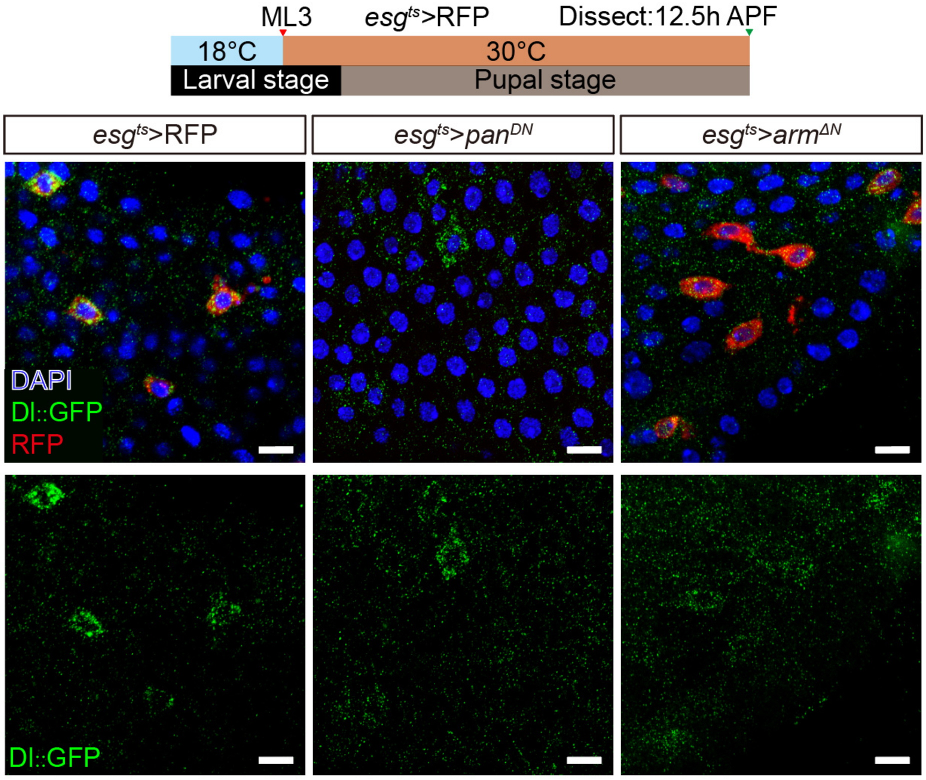

Fig. S8. Wnt signaling does not directly activate Dl expression.
Representative images of ISCs (*esg>RFP*, red) and *Dl* (*Dl*::GFP, green) staining in control (*esg*^{ts}*>RFP*), *esg*^{ts}*>pan*^{DN}, and *esg*^{ts}*>arm*$^{\Delta N}$ midguts at 12.5h APF. Wnt signaling manipulations was induced from ML3.

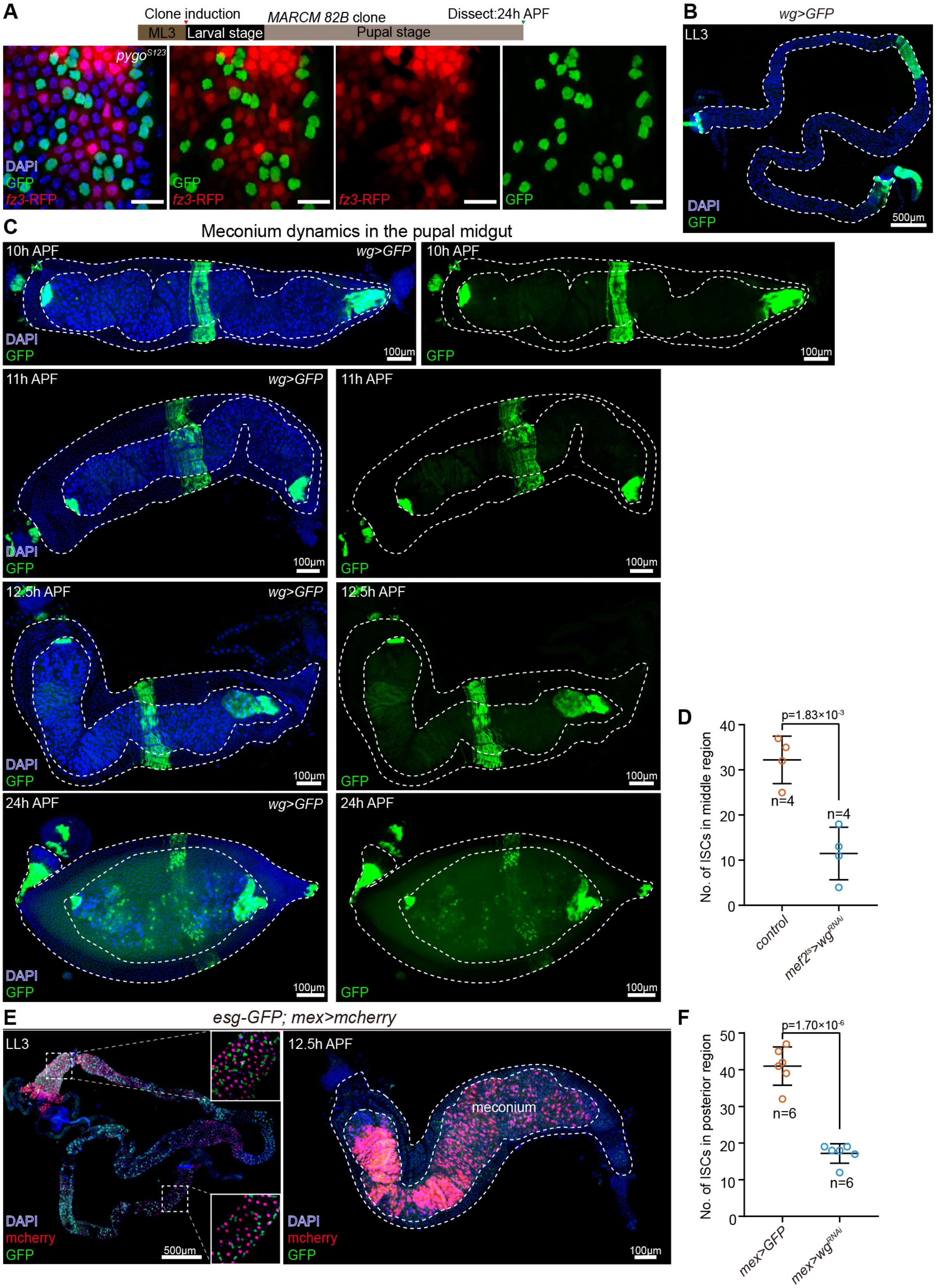

A
Clone induction
MARCM 82B clone
Dissect:24h APF
ML3
Larval stage
Pupal stage
pygo S123
DAPI
GFP
fz3-RFP
B
wg>GFP
LL3
500μm
C
Meconium dynamics in the pupal midgut
10h APF
11h APF
12.5h APF
24h APF
100μm
D
p=1.83×10-3
No. of ISCs in middle region
n=4
control
mef2ts>wgRNAi
E
esg-GFP; mex>mcherry
mcherry
meconium
F
p=1.70×10-6
No. of ISCs in posterior region
n=6
mex>GFP
mex>wgRNAi

Fig. S9. The meconium epithelium derived from larval ECs is the source of pupal midgut Wg.

(A) Representative images of *fz3*-RFP staining (red) in *pygo* mutant MARCM clones (GFP, green) at 24h APF. **(B)** Representative images of *wg*>GFP expression in the LL3 midgut. **(C)** Representative images showing the dynamic changes in Wg expression (*wg>GFP*) in the midguts at indicated developmental time points. Outer dashline show the outline of the midguts, while inner dashline show the outline of the meconium. **(D)** Statistics of ISC number in the middle region of *control* (*esg-GFP; Mef2^{ts}>attp2*) and *esg-GFP; Mef2^{ts}>wg^{RNAi}* midguts at 12.5h APF. n, number of mid midguts. The ISC number was counted in the middle region of approximately 200um width in the 12.5h APF midgut. **(E)** Representative images of *mex*>mCherry expression (red) and *esg*-GFP staining (green) in LL3 (left) and 12.5h APF (right) midguts. Inset represents close-up views in selected box regions. **(F)** Statistics of ISC number in the posterior region of *control* (*esg-GFP; mex>attp2*) and *esg-GFP; mex>wg^{RNAi}* midguts at 12.5h APF. n, number of midguts. Data are presented as mean ± SD. All images are representative of ≥20 biological replicates. Statistical analysis by two-tailed unpaired t-test. Scale bars, 20 µm unless otherwise specified.

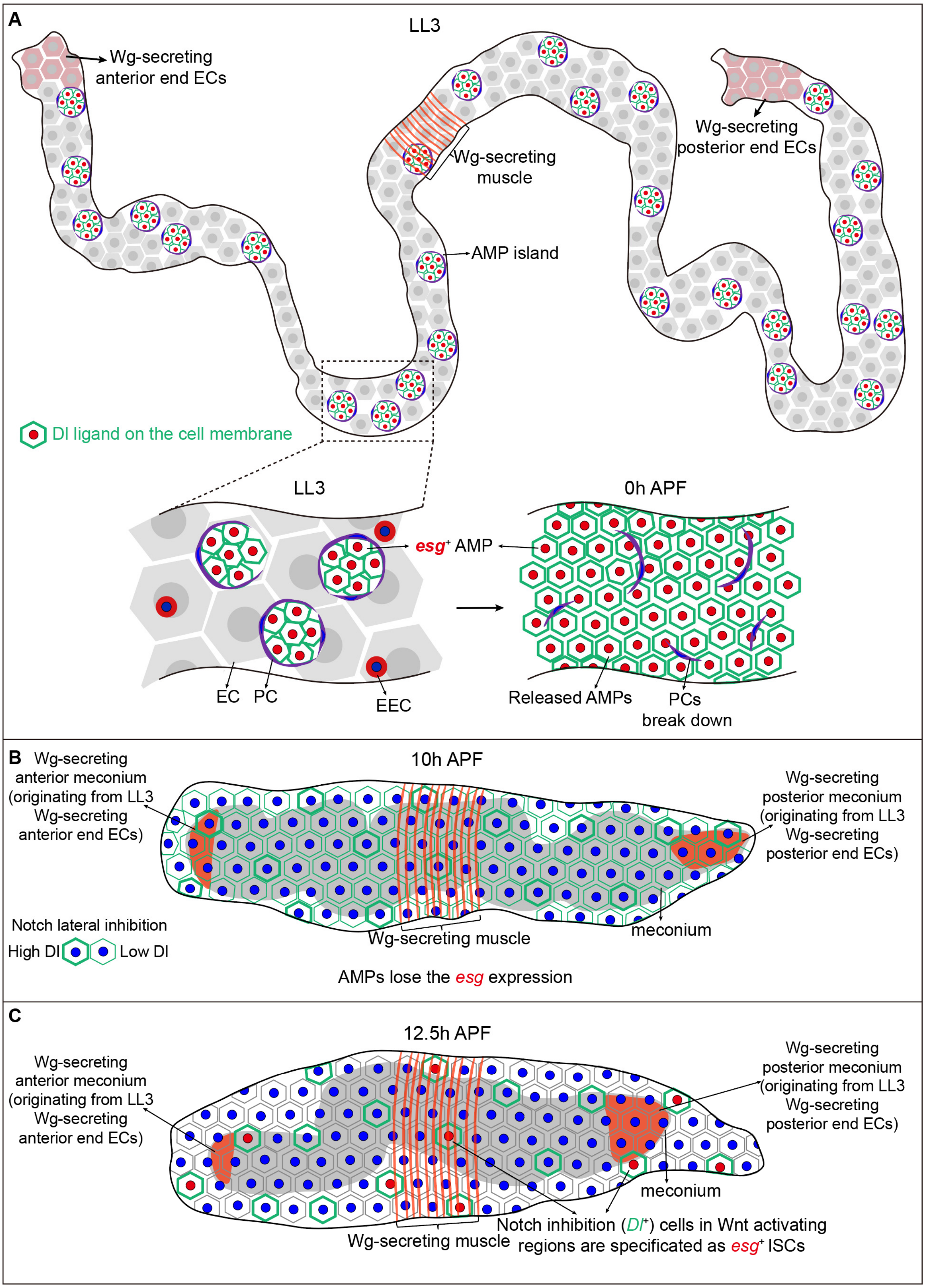
A
LL3
Wg-secreting
anterior end ECs
Wg-secreting
muscle
Wg-secreting
posterior end ECs
AMP island
Dl ligand on the cell membrane
LL3
0h APF
esg+ AMP
EC
PC
EEC
Released AMPs
PCs
break down
B
Wg-secreting
anterior meconium
(originating from LL3
Wg-secreting
anterior end ECs)
10h APF
Wg-secreting
posterior meconium
(originating from LL3
Wg-secreting
posterior end ECs)
Notch lateral inhibition
High Dl
Low Dl
Wg-secreting muscle
meconium
AMPs lose the esg expression
C
12.5h APF
Wg-secreting
anterior meconium
(originating from LL3
Wg-secreting
anterior end ECs)
Wg-secreting
posterior meconium
(originating from LL3
Wg-secreting
posterior end ECs)
meconium
Notch inhibition (Dl+) cells in Wnt activating
regions are specificated as esg+ ISCs
Wg-secreting muscle

Fig. S10. Model of ISC specification from larval AMP islands to the early pupal midgut. AMP: adult midgut progenitor; PC: peripheral cell; EC: enterocyte; EEC: enteroendocrine cell. **(A)** Late third instar larval stage (LL3): Dl is expressed in all AMPs within AMP islands, each surrounded by PCs. Wg is secreted by circular muscles in the midgut middle region and by ECs at the anterior and posterior ends. Upon pupariation (0h APF), larval ECs and EECs encapsulate the larval food to form the meconium. The anterior and posterior ends of the meconium continue to express Wg. PCs degenerate, and AMPs coalesce to form a uniform pupal midgut epithelium, with Dl initially expressed throughout. **(B)** ~10h APF: Dl expression becomes downregulated in most epithelial cells, remaining elevated only in scattered cells. Wnt signaling is activated in discrete anterior, middle, and posterior domains of the gut epithelium under the influence of Wg from muscle and meconium. **(C)** By 12.5h APF: Cells maintaining high Dl expression and located within Wnt-active zones are specified as ISCs.

Data S1 - Key resources: Fly and mouse strains used in the work

Experimental models: Organisms/strains	Resource	Stock number
esg-Gal4	Obtained from B.Ohlstein lab (*23*)	N/A
tub-Gal80^{ts}	Obtained from B.Ohlstein lab (*23*)	N/A
UAS-GFP	Obtained from B.Ohlstein lab (*23*)	N/A
esg-Gal4, tub-Gal80^{ts}, UAS-GFP	Obtained from B.Ohlstein lab (*23*)	N/A
attp2	BDSC	BL#36303
w^{1118}	Vienna *Drosophila* Resource Center (VDRC)	VDRC#6000
UAS-Cdk2^{RNAi}	Tsinghua Fly Center	THU201500107.s
esg-Gal4 tub-Gal80^{ts} UAS-GFP; UAS-flp Act>CD2>Gal4	Obtained from B.Ohlstein lab (*53*)	N/A
MARCM 82B	Obtained from B.Ohlstein lab	N/A
FRT82B	BDSC	BL#2035
MARCM 19A	Obtained from B.Ohlstein lab	N/A
FRT19A	BDSC	BL#1709
UAS-hid	Obtained from B.Ohlstein lab	N/A
Dl::GFP	Obtained from ZZ. Zhai lab (*47, 100*)	N/A
UAS-Notch^{RNAi}	BDSC	BL#7078
Mef2-Gal4	Obtained from X. Huang lab (*101*)	N/A
vm-Gal4	BDSC (*102*)	BL#48547

UAS-Notch^{intra1790}	Obtained from G Struhl lab (*54*)	
UAS-Dl^{RNAi}	BDSC	BL#34322
Dl-Gal4 UAS-GFP	Obtained from Steven X. Hou (*48*)	N/A
UAS-Raf	BDSC	BL#2033
UAS-Ras85D^{RNAi}	Obtained from Bruce Edgar lab (*71*)	N/A
Tet-EGFP	Obtained from RJ. Tu lab (*35*)	N/A
UAS-arm$^{\Delta N}$	Obtained from G Struhl lab (*60*)	N/A
UAS-dsh^{RNAi}	BDSC	BL#31307
UAS-wg-HA (II)	Obtained from ZZ. Zhai lab	N/A
W; UAS-wg-3XHA (III)	BDSC	BL#5918
FRT82B APC1^{Q8}*APC2*$^{19.3}$	Obtained from Y. Ahmad lab (*67, 68*)	N/A
FRT82B APC1^{Q8}	Obtained from Y. Ahmad lab (*66*)	N/A
w [+]/w; fz3-RFP; FRT82Bpygo^{s123}*/TM6.sb*	Obtained from Y. Ahmad lab (*76*)	N/A
wg {KO; Gal4}	Obtained from Vincent JP lab (*77*)	N/A
esg-LacZ	BDSC	BL#10359
UAS-wg^{RNAi}	Tsinghua Fly Center	THU1187
mex-Gal4, esg-GFP/cyo; UAS-mcherry	Obtained from HY. Chen lab (*103*)	N/A
esg-GFP	BDSC	BL#78333
UAS-Egfr^{RNAi}	Obtained from K. Irvine Lab	VDRC#107130
UAS-arm^{RNAi}	BDSC	BL#35004
Mouse: *Lgr5-EGFP-IRES-CreERT2*	Shanghai Model Organisms Center, Inc..	NM-KI-200154

Other Supplementary Materials

Table S1.
A list of differentially expressed genes and marker genes in each cluster.

Table S2.
Gene Ontology enrichment of cluster 5 and 6.

Table S3.
Gene Ontology enrichment of cluster 0.

Table S4.
A list of five classes of differential genes clustered at the nodes of the two cell fates of differentiated AMPs.

Table S5.
A list of differential genes clustered at the nodes of fate specification of differentiated AMP cells toward ISC-p.
